# AHAS structural changes caused by mutations result in differences in binding affinity and resistance to two herbicides in *Cyperus difformis* L.

**DOI:** 10.3389/fpls.2026.1932845

**Published:** 2026-09-17

**Authors:** Xiaotong Guo, Xiangju Li, Jingchao Chen, Linzhi Bai, Haiyan Yu, Hailan Cui

**Affiliations:** 1 State Key Laboratory for Biology of Plant Diseases and Insect Pests, Institute of Plant Protection, Chinese Academy of Agricultural Sciences, Beijing, China; 2 Institute of Plant Protection, Heilongjiang Academy of Agricultural Sciences, Heilongjiang, Harbin, China

**Keywords:** affinity, *Cyperus difformis*, molecular docking, mutation, prokaryotic expression

## Abstract

As a malignant weed in paddy systems, *Cyperus difformis* L. (*C. difformis*) has evolved resistance to acetohydroxyacid synthase (AHAS, EC 2.2.1.6), also known as acetolactate synthase (ALS), inhibiting herbicides such as pyrazosulfuron-ethyl and bensulfuron-methyl due to prolonged selection pressure. This study systematically investigated the interaction mechanisms between mutated AHAS and these herbicides through biochemical and structural approaches. In this study, purified AHAS proteins of five mutated types and the wild type were obtained via prokaryotic expression, and their sensitivity and affinity for pyrazosulfuron-ethyl and bensulfuron-methyl were determined. The structural differences in different binding of AHAS binding to the two herbicides were identified via homology modeling and molecular docking. The sensitivity determination of purified AHAS indicated that AHAS with site 197 mutations had higher GR_50_ and I_50_ values for pyrazosulfuron-ethyl (25.60–74.78 μM) than for bensulfuron-methyl (6.62–38.32 μM). The AHAS with a site 376 mutation had higher GR_50_ and I_50_ values for bensulfuron-methyl than for pyrazosulfuron-ethyl, while the GR_50_ and I_50_ values of the AHAS with a site 574 mutation were the highest. The affinity of purified AHAS for the substrate revealed that all the mutations increased the affinity except for the Pro-197-Arg mutation. In addition, all the AHAS mutants had greater affinity for bensulfuron-methyl than for pyrazosulfuron-ethyl. Molecular docking results revealed that AHAS formed hydrogen bonds, π–π bonds, and hydrophobic interactions with pyrazosulfuron-ethyl and bensulfuron-methyl. The total number and types of interactions with pyrazosulfuron-ethyl and benzosulfuron-methyl were obviously different between the mutant AHAS and wild-type AHAS. Therefore, in this study, different mutations in AHAS induced distinct structural alterations, which reduced the affinity of the enzyme for herbicides, consequently leading to the development of weed resistance. The difference in affinity between the same mutant and the two herbicides led to a difference in resistance to the two herbicides. The results of this study are helpful for understanding the causes of weed resistance to herbicides at the structural level.

## 1 Introduction

*C. difformis*, an annual weed of the Cyperaceae family, is a malignant weed in rice fields. Weeds have adapted to ecologic conditions for rice growth because of natural selection during agricultural production. As the most widely used herbicide target, AHAS is a flavoprotein encoded by nuclear genes that is present in the chloroplasts of higher plants (Yu and Powles, 2014). AHAS is a key enzyme in the synthesis of branched-chain amino acids (leucine, valine, and isoleucine) in plants and microorganisms (Duggleby et al., 2008). AHAS inhibitors can interfere with the synthesis of branched-chain amino acids to prevent the synthesis of corresponding proteins in plants by inhibiting AHAS activity, leading to the inhibition or death of plants.

Resistant weeds are currently present in 102 crops in 76 countries worldwide and are resistant to approximately 168 herbicides across 21 different sites of action (Heap, 2026). Weed resistance can be divided into target-site resistance and non-target-site resistance according to previous reports (Rey-Caballero et al., 2017; Lu et al., 2019; Gaines et al., 2020; Amrhein and Martinoia, 2021). Preliminary studies revealed that the resistance mechanisms of weeds to AHAS inhibitors are due primarily to AHAS mutations (Tehranchian et al., 2001, Tehranchian et al., 2015; Ntoanidou et al., 2016; Choudhary et al., 2021; Yin et al., 2023). For AHAS inhibitors, resistance in weeds is often caused by amino acid substitutions in the AHAS enzyme that alter herbicide binding and thereby reduce sensitivity to sulfonylurea, imidazolinone, and related herbicides (Yu et al., 2023). Several highly conserved residues in or near the substrate access channel of AHAS/ALS, including Pro197, Asp376, and Trp574, are key determinants of herbicide binding. Substitutions at these positions frequently reduce herbicide affinity by changing the 3D structure of the enzyme and, in some cases, the local interaction network within the binding pocket, thereby conferring target-site resistance (McCourt et al., 2006; Zhang et al., 2017). Resistance to cloransulam methyl, chlorimuron-ethyl, and bispyribac-sodium in *Conyza canadensis* L. has been attributed to Pro197Ser/Ala and Asp376Glu mutations in the AHAS gene (Zheng et al., 2011). In *C. difformis*, the AHAS mutations identified so far are mainly single amino-acid substitutions at positions 197, 376, and 574 (e.g., Pro197Ser/Arg/Leu/His/Ala, Asp376Glu, and Trp574Leu), while a few studies have also reported variants such as Pro97His and Ala122Asn (Tehranchian et al., 2015; Ntoanidou et al., 2016; Li et al., 2020; Ceseski et al., 2023; Guo et al., 2024). The ploidy level of *C. difformis* has not yet been clearly and consistently reported (Li et al., 2020, Li et al., 2026). In addition, AHAS with the Trp574Leu mutation confers resistance to multiple herbicides in *Amaranthus palmeri* S. Watson (Larran et al., 2017). Mutations in AHAS may affect its affinity for substrates and lead to different resistance levels (Han et al., 2012; Li et al., 2013; Fang et al., 2022). The *Km* value represents the affinity of AHAS for substrates. AHAS with the Pro197Ser mutation resulted in a decrease in the *Km* in *Stellaria aquatica* L (Liu et al., 2017). The *Km* of AHAS with the Pro197Ala/Gln and Trp574Leu mutations also decreased in *Lolium perenne* L. and *Alopecurus aequalis* Sobol, respectively (Yu et al., 2010; Zhao et al., 2020). However, AHAS with the Pro197Arg/Tyr mutation led to a relatively high *Km* in *Lolium perenne* L (Yu et al., 2010). AHAS with the Trp574 mutation changes the shape of the binding site between herbicides and AHAS, endowing weeds with resistance to herbicides (Yang et al., 2018).

In this study, analyzing the weed resistance mechanism from the perspective of protein structure can elucidate the specific mechanisms by which weeds develop resistance to herbicides. Certain weeds may enhance resistance through alterations in the structure of proteins, particularly those associated with herbicide target genes. Investigating these structural modifications can provide insights into the molecular basis of weed resistance and offer valuable references for the design of novel herbicides. Additionally, examining the variations in protein structure across different weed populations can help explain the observed differences in resistance levels. Some weed populations exhibit distinct protein structural changes that result in varying degrees of resistance to the same herbicide. By comparing the protein structures of weed populations with different resistance levels, we can gain a deeper understanding of the molecular mechanisms underlying these differences, thereby providing a theoretical foundation for further research on the development and management of weed resistance.

## 2 Materials and methods

### 2.1 Plant materials

The seeds of *C. difformis* were collected, and the biotypes resistant to bensulfuron-methyl were screened via a whole-plant dose–response bioassay. The *AHAS* genes of different resistant biotypes were detected to confirm mutation types. Seeds from each individual plant with a homozygous mutation were isolated and bagged individually for propagation. The obtained seeds were propagated for two generations, and the *AHAS* genes were detected from five randomly selected plants in each generation to ensure seed purity. The susceptible biotype was also detected via the same method and propagated for two generations. The plant material contained five mutations (Pro197Leu, Pro197Ser, Pro197Arg, Asp376Glu, and Trp574Leu) and one nonmutated susceptible population (wild type).

The nutrient soil and organic fertilizer were mixed at a ratio of 3:1, and a pot with a diameter of 9 cm × 9 cm × 11 cm was filled. The seeds were soaked in ethephon at a concentration of 50 mg/kg for 12–14 h and sown in the pots. The seedlings were cultivated in a greenhouse with 12 h/12 h day/night light cycle.

### 2.2 Whole-plant dose-response bioassay

The plants were treated at the 3–4-leaf stage via a moving TeeJet^®^ XR8002 flat fan nozzle cabinet sprayer at a pressure of 0.275 Mpa, with a spray volume of 450 L·ha^−1^ (Compressed Air Cabinet Sprayer ASS-4, Beijing Research Center for Information Technology in Agriculture, Beijing, China). Different doses of bensulfuron-methyl were sprayed on *C. difformis* with three replicates for each treatment (Appendix 1). After 21 days of herbicide treatment, the aboveground plants were cut and dried at 70 °C to a constant weight. The GR_50_ values for dry weight inhibition after treatment with bensulfuron-methyl were calculated using a double-logistic nonlinear regression model via SigmaPlot (*v.*12.5) according to Equation 1, where b denotes the slope of the curve and *Y* represents the dry weight inhibition rate under herbicide treatment. *D* and *C* denote the upper and lower limits of the dose–response curves, respectively. *X* represents the herbicide dose, and GR_50_ represents the herbicide dose required to reduce the dry weight by half.

(1) $$Y=C+\frac{D-C}{1+{\left(\frac{X}{GR_{50}(I_{50})}\right)}^{b}}$$

### 2.3 *In vitro* AHAS activity assay

Leaf tissue (4.5 g) was taken from each biotype at the 4–5-leaf stage and stored at -80 °C. Different AHAS mutants were extracted and separately mixed with different concentrations of bensulfuron-methyl. The amount of pyruvate produced after 30 min of reaction at 37 °C was measured according to the method described by Li et al (Li et al., 2020). The concentrations of bensulfuron-methyl for the wild-type and mutant AHAS were 0, 0.01, 0.1, 1, 10, 100, 1000, 10000 μM, respectively. The data were analyzed via the nonlinear regression model in Sigmaplot (*v.*12.5), and the I_50_ was calculated based on Formula (1), where I_50_ represents the concentration of herbicide required for 50% inhibition of AHAS activity (Chen et al., 2015).

Different purified AHAS mutants were reacted with various concentrations of pyrazosulfuron-ethyl and bensulfuron-methyl, which was consistent with the above concentration settings. The measurement method was previously described by Li et al (Li et al., 2020). The experiment was repeated three times with three replicates each time. The data was analyzed for significance using one-way ANOVA in SPSS (v.19.0).

### 2.4 AHAS protein prokaryotic expression and purification

Using the amino acid sequence of AHAS from *C. difformis* as a template, cDNA was synthesized and codon optimized for *Escherichia coli*. The optimized DNA sequence was cloned and inserted into the BamHI (5’) and XhoI (3’) restriction sites of the pET-28a expression vector (New England Biolabs Inc., Ipswich, MA, USA). The transformed plasmid was confirmed by sequencing, and successful constructs were stored at -80 °C in cryotubes with a 1:1 ratio of 40% glycerol and bacterial culture. The pET-28a plasmid containing the target gene was transformed into RIPL competent cells, which were subsequently cultured on LB agar plates (Kan). The cells were induced with 0.5 mM IPTG at 37 °C for 4 h (200 rpm, OD_600_ = 0.8) and centrifuged at 3000 rpm for 30 min. The bacterial pellet was resuspended in 20 mM Tris and 300 mM NaCl at a pH of 7.4, and 0.1% PMSF was added to the suspension. The suspension was sonicated and centrifuged at 13,000 rpm for 25 min, and the supernatant was filtered through a 0.22 μm membrane. The filtered supernatant was loaded onto the AKTA advant at a flow rate of 1–1.6 ml/min. The target protein was eluted from the column with elution buffer containing 0.2 M imidazole for 5–10 CV (column volumes). The eluted samples induced by *Escherichia coli* were analyzed via SDS–PAGE. The purified target protein was desalted via a PD10 column (GE) with buffer containing 20 mM Tris and 300 mM NaCl (pH 7.4). The protein concentration was determined via a NanoDrop with the protein 280 assay, and the measurement was compared with a standard curve generated via BSA and other proteins (E 1%). The dye reagents used to determine the protein concentration were obtained from Bio-Rad Laboratories Inc. (Hercules, CA, USA).

### 2.5 Detection of kinetic parameters of purified AHAS

Different purified AHAS mutants were reacted with different concentrations of sodium pyruvate to detect kinetic parameters. The measurement method was previously described (Yu et al., 2010) with slight modifications, where no sodium pyruvate was added to the reaction buffer. The concentration of sodium pyruvate solution was set to 0.39–25 mm. The kinetic parameters *Km* and *Vmax* were calculated via the Michaelis–Menten kinetic equation: *v* = *Vmax* [*S*]/(*Km* + [*S*]). The experiment was repeated three times, with three replicates each time. The data was analyzed for significance using one-way ANOVA in SPSS (v.19.0).

### 2.6 Affinity detection between purified AHAS and herbicides

The mutant and wild type AHAS were exchanged via the PD10 column (17-0851-01) with PBS buffer according to the manufacturer’s instructions. The concentration was concentrated to 5–10 mg/ml so that it could be coupled with streptavidin. A 30 kd ultrafiltration tube (Merck KGaA, Darmstadt, Germany) was used and pretreated with ultrapure water. The samples collected from the above step were added to the inner tube of the ultrafilter and centrifuged at 2500 rpm for 20 min. The concentration of the target protein was detected via the Bio-Rad Protein Assay Kit I (500-0001) according to the Bradford method (Kumar et al., 2019). The target protein was coupled with biotin via an EZ-Link™ Sulfo-NHS-LC-biotinylation kit (Thermo Fisher: 21435). Biotin was added to the target protein at a concentration ratio of 20:1 with Sulfo-NHS-LC-biotin, and the reaction was carried out at room temperature for 2 h. The affinity between the biotinylated target protein and small molecules was measured via the ForteBio Octet 96 system with super streptavidin (SSA) biosensors (18-5057) (Abdiche et al., 2008). The target protein was diluted in HEPES buffer to a concentration of 200 μg/mL, and biocytin was diluted 500 times. The SSA sensors were soaked in 50 mM HEPES buffer (pH 7.5) for 10 min for activation. The machine’s detection parameters were provided in Attachment 1. The double subtraction method, including subtracting the background solution and subtracting the blank sensor background, was used to determine the affinity between the target protein and small molecules. The original data were analyzed and processed via ForteBio analysis software to obtain the corresponding analysis results.

### 2.7 Homologous modeling and molecular docking

Homology modeling was performed via SWISS-MODEL (https://swissmodel.expasy.org/), and the constructed model was evaluated via Verify 3d, Procheck, and Errat in SAVES (v.6.0) (https://saves.mbi.ucla.edu/usage). Verify 3d can be used to evaluate the quality of a 3D model by analyzing the distribution of the structural types of amino acid residues, including *α*-helix, *β*-turn, loop, polar and nonpolar. A model with more than 80% residues having a 3D-1D ≥ 0.2 can be considered a high-quality model (Eberlein et al., 1997). The stereochemical quality of the protein model was assessed via Procheck by analyzing the angles between residues and positions in the overall structure. The Ramachandran plot can be used to determine the number and ratio of residues in the most favored regions, additional allowed regions, generously allowed regions, and disallowed regions, which are used to evaluate the quality of the model (Laskowski et al., 1993). Errat calculates a score by analyzing the statistical data of various atomic interactions, and the model can be considered high quality when the score exceeds 50 (Colovos and Yeates, 2010). The docking sites of several AHAS binding models with sulfonylurea inhibitors reported in the PDB database (https://www.rcsb.org/) were used to confirm the docking region in this study and remove redundant ligands from the established model. The 3D structures of the two herbicides were downloaded from the ChemSpider database (http://www.chemspider.com/), and energy minimization and hydrogenation were performed. The processed protein and herbicide molecules were semiflexibly docked via AutoDock.

## 3 Results

### 3.1 The resistance levels of *C. difformis* with different mutations to bensulfuron-methyl

The GR_50_ of the susceptible biotype YX15–30 was 0.88 g a.i. ha^-1^ on the basis of the whole-plant bioassay. The resistance indices of the five mutant biotypes were in the range of 461.1-1636.0 (**Table 1**). The resistance level of YX15-37-1 (Trp574Leu) to bensulfuron-methyl was the highest. The resistance level of YX13-17-1 (Asp376Glu) was higher than those of the three biotypes with 197 mutations, while the latter had a lower resistance level to pyrazosulfuron-ethyl (Guo et al., 2024).

**Table 1:** The GR_50_ and I_50_ to bensulfuron-methyl in *C. difformis*.

Populations	Mutations	GR_50_ ^a^ (SE) (g a.i. ha^-1^)	Resistance index	I_50_ ^b^ (SE) (μM)	Resistance index
YX15-30	Wild type	0.88 (0.15)	—	0.037 (0.01)	—
YX15-24-1	Pro197Arg	405.76 (23.41)	461.1	15.47 (4.15)	418.1
YX15-22-1	Pro197Leu	248.33 (16.30)	282.2	3.75 (1.32)	101.4
YX15-22-3	Pro197Ser	573.84 (134.34)	652.1	20.88 (3.98)	564.3
YX13-17-1	Asp376Glu	1439.71 (61.95)	1636.0	59.07 (6.09)	1596.5
YX15-37-1	Trp574Leu	>7680.00	—	285.15 (42.58)	7706.8

^a^ GR_50_, the herbicide dose required to reduce the dry weight by half.

^b^ I_50,_ represents the concentration of herbicide required for 50% inhibition of AHAS activity.

### 3.2 Sensitivity of AHAS with different mutations to bensulfuron-methyl

The sensitivity of AHAS to bensulfuron-methyl indicated that the I_50_ of the susceptible AHAS was 0.037 μM. The I_50_ values of the five AHAS mutants were 3.75-285.15 μM (**Table 1**). The sensitivity of AHAS mutants to bensulfuron-methyl was significantly decreased, and the sensitivity of the Trp574Leu mutant was the lowest. The Pro197Ser/Leu/Arg mutation resulted in greater sensitivity to bensulfuron-methyl than did the Asp376Glu mutation, while the latter was more sensitive to pyrazosulfuron-ethyl (the sensitivity to pyrazosulfuron-ethyl has been published) (Guo et al., 2024).

### 3.3 The sensitivity of purified AHAS to herbicides and AHAS kinetic parameters

The six different biotypes of AHAS were successfully expressed in *Escherichia coli*, and the concentrations of the purified proteins are shown in **Table 2**. The sensitivity and kinetic parameters of purified AHAS to pyrazosulfuron-ethyl and bensulfuron-methyl were detected. The sensitivity determination results revealed that the mutated AHAS exhibited decreased sensitivity to both pyrazosulfuron-ethyl and bensulfuron-methyl, and the Trp574Leu mutation resulted in the lowest sensitivity. Compared with the Asp376Glu mutation, the Pro197Ser/Leu/Arg mutation resulted in lower sensitivity to pyrazosulfuron-ethyl but greater sensitivity to bensulfuron-methyl (**Figure 1**). The kinetic data of AHAS revealed that the *Km* values of the Asp376Glu, Pro197Leu, Trp574Leu, and Pro197Ser mutants were lower (0.43**–**0.98 times) than those of the susceptible AHAS. The affinity of the above AHAS mutants for the substrate increased, but the *Km* of AHAS with the Pro197Arg mutation increased, indicating that its affinity was reduced (**Table 3**). The *Vmax* values of the five AHAS mutants were 1.09**–**5.27 times greater than that of the susceptible AHAS.

**Table 2:** I_50_ values to pyrazosulfuron-ethyl and bensulfuron-methyl in *C. difformis*.

Mutations	Pyrazosulfuron-ethyl			Bensulfuron-methyl		
	I_50_ ^a^ (SE) (μM)	Resistance index	Correlation coefficient	I_50_ (SE) (μM)	Resistance index	Correlation coefficient
Wild type	0.12 a	—	1.00	0.04 a	—	1.00
Asp376Glu	18.31 b	152.6	1.00	178.98 e	4474.5	1.00
Pro197Leu	25.60 b	213.3	0.99	6.62 b	165.5	1.00
Pro197Ser	74.78 c	623.2	1.00	38.32 d	958.0	1.00
Pro197Arg	65.83 c	548.6	1.00	23.83 c	595.8	1.00
Trp574Leu	152.94 d	1274.5	0.99	191.39 f	4784.8	1.00

^a^ I_50_, the concentration of herbicide required for 50% inhibition of AHAS activity.

Different letters indicate significant differences (P<0.05).

**Figure 1:**
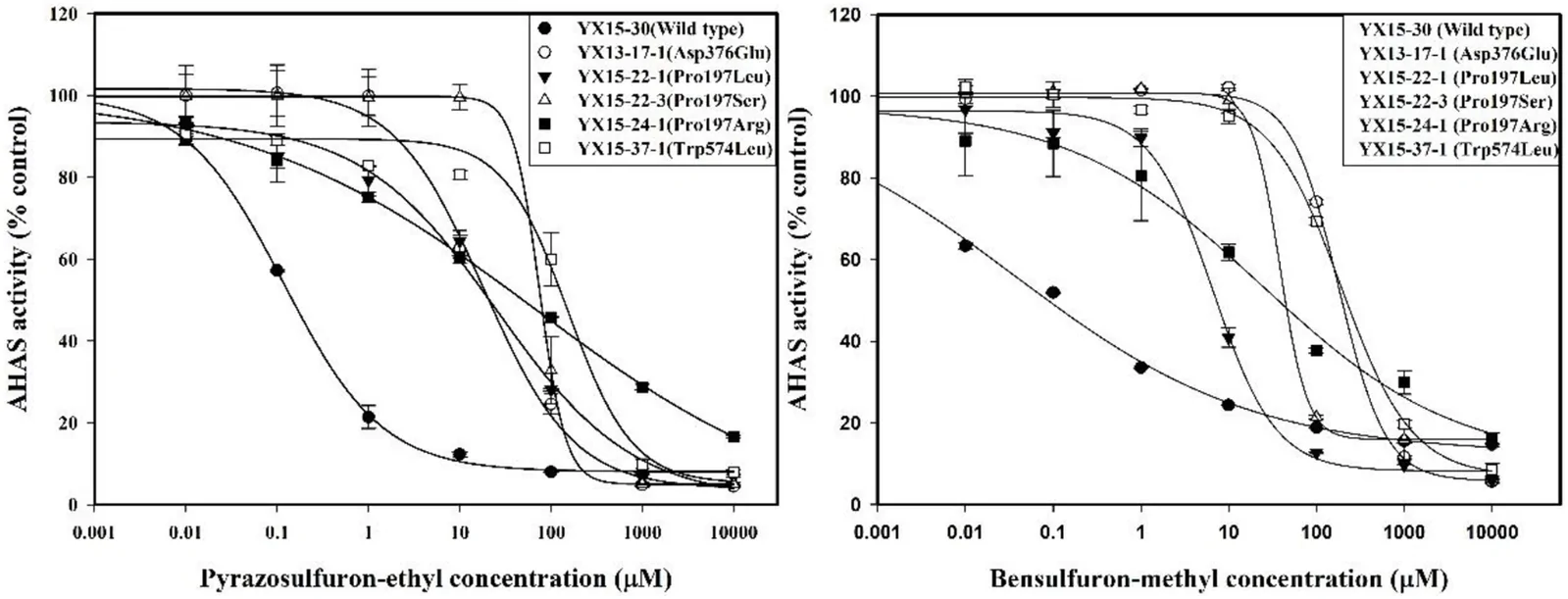
Dose–response curves of purified AHAS with different mutations to pyrazosulfuron-ethyl and bensulfuron-methyl.

**Table 3:** *Km* and *Vmax* values of different mutant AHAS.

Mutations	*Km* (mM) ^a^	R/S	*Vmax* (nmol mg^-1^ min^-1^) ^b^	R/S
Wild type	9.12 c	—	0.11^a^	—
Asp376Glu	3.95 a	0.43	0.58^e^	5.27
Pro197Leu	6.37 b	0.70	0.19^b^	1.73
Pro197Ser	8.95 c	0.98	0.32^c^	2.91
Pro197Arg	10.21 d	1.12	0.12^a^	1.09
Trp574Leu	3.71 a	0.41	0.42^d^	3.82

^a^ *Km*, Michaelis-Menten constant, indicated the affinity between AHAS and the substrate.

^b^ *Vmax*, the reaction rate of AHAS when it is completely saturated with the substrate.

Different letters indicate significant differences (P<0.05).

### 3.4 Affinity of purified AHAS with pyrazosulfuron-ethyl and bensulfuron-methyl

The concentrations of the six purified AHASs are displayed in Appendix 1. The binding affinities of the six biotypes of AHAS with pyrazosulfuron-ethyl and bensulfuron-methyl were measured via the Fortebio Octet system (**Figure 2**; **Table 4**). The affinity was evaluated according to the formula *Kd*=*Koff*/*Kon*, where *Kon* represents the association rate of small molecules with proteins, *Koff* denotes the dissociation rate of small molecules from proteins, and a larger *Kd* indicates a weaker affinity between the small molecule and protein. The results indicated that the *Kd* (molecular concentration: 0.01 M) of AHAS with pyrazosulfuron-ethyl was significantly greater than that of bensulfuron-methyl (molecular concentration: 0.1 mM). The results revealed that the affinities between pyrazosulfuron-ethyl and the six AHASs were weaker than those of bensulfuron-methyl, which may have contributed to the greater inhibitory effect of bensulfuron-methyl than that of pyrazosulfuron-ethyl. The affinities between AHAS mutants and the two herbicides were significantly different from those of the susceptible AHAS, indicating that the mutations significantly changed the affinity between AHAS and the herbicides.

**Figure 2:**
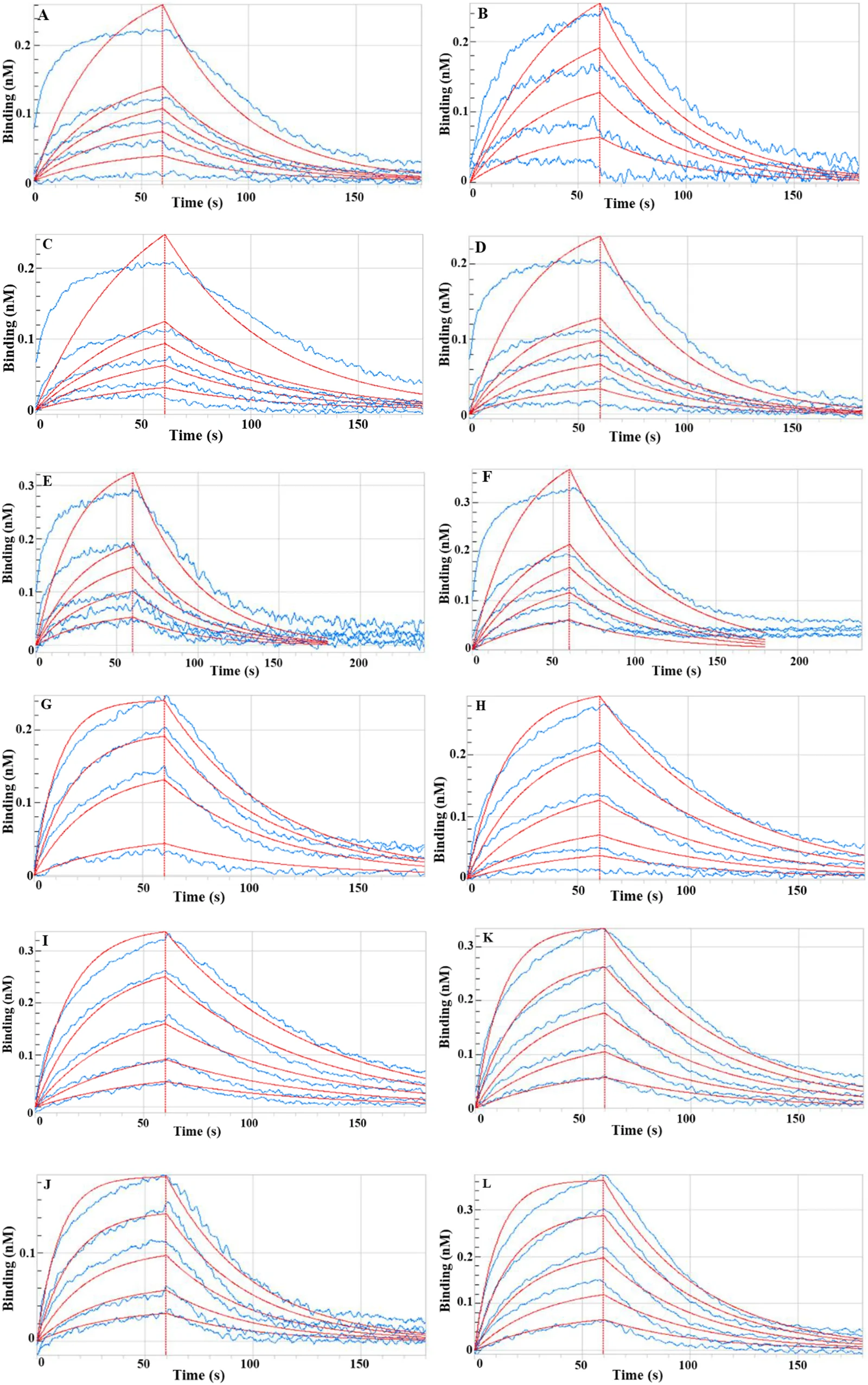
Binding analysis of six AHASs with pyrazosulfuron-ethyl and bensulfuron-methyl via the ForteBio Octet system with high-capacity super streptavidin (SSA) biosensors. **(A)**, Wild type to pyrazosulfuron-ethyl; **(B)**, Asp376Glu to pyrazosulfuron-ethyl; **(C)**, Pro197Leu to pyrazosulfuron-ethyl; **(D)**, Pro197Ser to pyrazosulfuron-ethyl; **(E)**, Pro197Arg to pyrazosulfuron-ethyl; **(F)**, Trp574Leu to pyrazosulfuron-ethyl. **(G)**, Wild type to bensulfuron-methyl; **(H)**, Asp376Glu to bensulfuron-methyl; **(I)**, Pro197Leu to bensulfuron-methyl; **(J)**, Pro197Ser to bensulfuron-methyl; **(K)**, Pro197Arg to bensulfuron-methyl; **(L)**, Trp574Leu to bensulfuron-methyl.

**Table 4:** The binding affinities of the six AHASs for pyrazosulfuron-ethyl and bensulfuron-methyl.

Mutations	Pyrazosulfuron-ethyl			Bensulfuron-methyl		
	*KD (M) ^a^*	*Kon(M^-1^S^-1^) ^b^*	*Koff (S^-1^) ^c^*	*KD (M)*	*Kon (M^-1^S^-1^)*	*Koff (S^-1^)*
Wild type	5.036E-02	0.5228	0.02633	4.406E-04	45.01	0.01983
Asp376Glu	7.620E-01	0.03453	0.02631	7.168E-04	23.97	0.01718
Pro197Leu	4.786E-01	0.04232	0.02025	5.096E-04	29.53	0.01505
Pro197Ser	4.637E-02	0.5973	0.0277	4.422E-04	39.99	0.01768
Pro197Arg	2.201E-02	1.195	0.02631	5.607E-04	45.11	0.02529
Trp574Leu	1.725E-02	1.133	0.01955	4.741E-04	47.41	0.02247

^a^ *KD*, dissociation constant.

^b^ *Kon*, association rate constant.

^c^ *Koff*, disassociation rate constant.

### 3.5 Binding analysis of different AHAS with pyrazosulfuron-ethyl and bensulfuron-methyl

The evaluation results of the six AHAS models (**Supplementary Table S5**) indicated that all the models were suitable for use in molecular docking (Appendix 1). The binding energies of AHAS with pyrazosulfuron-ethyl and bensulfuron-methyl are shown in **Table 5**. From the docking results, it was observed that the types and numbers of interactions—mainly hydrogen bonds, hydrophobic interactions, and π–π bonds—between mutant AHAS and the herbicides tended to be reduced compared with the wild type, which was accompanied by decreased predicted affinity and increased resistance levels (**Figure 3**). Different interaction patterns were also detected among various AHAS mutants when docked with the same herbicide, suggesting a possible association with differential resistance levels. For example, more hydrogen bonds were formed between the Asp376Glu mutant and pyrazosulfuron-ethyl than between the same mutant and bensulfuron-methyl (4 vs. 2), and this difference in predicted binding patterns may be correlated with a relatively higher resistance to bensulfuron-methyl. In addition, weaker predicted interactions were observed between the Pro197Arg/Ser/Leu mutants and pyrazosulfuron-ethyl compared with the Asp376Glu mutant, whereas the opposite trend was found for bensulfuron-methyl, which might be related to the differing resistance profiles between these mutants toward the two herbicides. However, it should be noted that molecular docking is only a predictive approach and cannot be used to establish a direct causal relationship between structural changes and biological resistance.

**Table 5:** Docking results of six AHAS with pyrazosulfuron-ethyl and bensulfuron-methyl.

Mutations	Pyrazosulfuron-ethyl			Bensulfuron-methyl		
	Binding energy (kcal mol^–1^)	Hydrogen bonds	Hydrophobic interactions	Binding energy (kcal mol^–1^)	Hydrogen bonds	Hydrophobic interactions
Wild type	-6.86	Arg377 (3) Lys256 (1)	Gly121, Val196, Pro197, Met200, Ala205, Phe206, Asp376, Met570, Trp574, Ser653	-6.76	Arg377 (2) Lys256 (1)	Gly121, Gln195, Val196, Pro197, Met200, Ala205, His352, Asp376, Arg377, Met570, Trp574
Asp376Glu	-6.50	Arg377 (3) Lys256(1)	Gly121, Val196, Pro197, Met200, Ala205, Phe206, Met570, Ser653	-6.12	Arg377 (1) Gln207 (1)	Phe206, Leu322, His352, Met490, Met570, Val542, Trp574
Pro197Leu	-6.47	Arg377 (1) Gln260 (1) Lys256(1)	Gly121, Ala122, Val196, Met200, Ala205, Trp574	-8.39	Arg377 (2) Lys256 (1)	Ala122, Val196, Met200, Phe206, Gln207, Met570, Ser653
Pro197Ser	-6.94	Arg377 (2) Gly121 (1)	Met200, Ala205, Phe206, Met570, Trp574, Lys256	-4.82	Arg377 (1) Gln207 (2)	Gly121, Val196, Met200, Lys256, Arg377, Met570, Trp574
Pro197Arg	-5.92	Arg377 (2) Gly121 (1)	Phe206, Lys256, Met513, Met570	-6.97	Arg377 (1) Gln207 (2)	Gly121, Ala122, Val196, Met200, Phe206, Met570, Trp574, Ser653
Trp574Leu	-5.77	Arg377 (2) Ser653 (1) Lys256 (1)	Ala122, Pro197, Phe206, Leu574, Met570	-5.66	Arg377 (1) Lys256 (1)	Val196, Pro197, Met200, Ala205, Trp574, Phe578, Ser653

**Figure 3:**
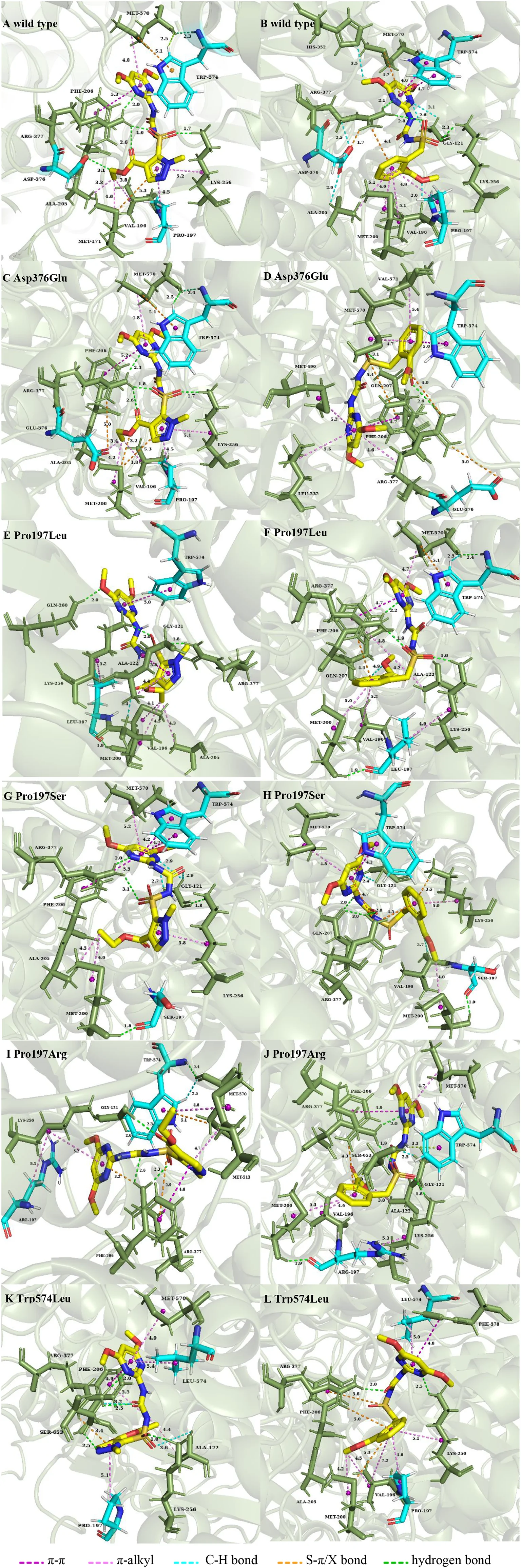
Docking results of different biotypes with pyrazosulfuron-ethyl **(A, C, E, G, I, K)** and bensulfuron-methyl **(B, D, F, H, J, L)**.

Pearson correlation analysis of the multi-parametric dataset revealed a strong correlation between pI_50_ and pGR_50_ (r = 0.992, p< 0.01), with other pairwise correlations being moderate to weak (Appendix **Supplementary Table S6**).

## 4 Discussion

AHAS with the Trp574Leu mutation presented the highest resistance to pyrazosulfuron-ethyl and bensulfuron-methyl in this study. High resistance caused by the Trp574Leu mutation has been reported in multiple weed species, including *Raphanus raphanistrum* L., *Rapistrum rugosum* L., *Descurainia sophia* L. and *Amaranthus palmeri* S (Yu et al., 2012; Larran et al., 2017; Zhang et al., 2017; Ntoanidou et al., 2018; Palmieri et al., 2022). Different mutations in AHAS have different effects on its affinity for the substrate. The Pro197Arg mutation resulted in a decrease in substrate affinity in this study, which is consistent with the affinity between AHAS and the substrate in *Alopecurus aequalis* Sobol (Liu et al., 2017). However, the affinity between the substrate and AHAS mutant was unchanged in some weeds. The Pro197Ser and Trp574Leu mutations had no significant effect on substrate affinity in *Lolium rigidum* L (Yu et al., 2010). The substrate affinity of AHAS with the Pro197Leu/Ser, Asp376Glu, and Trp574Leu mutations increased in this study, and the results were similar to those reported in *Descurainia sophia* L (Yang et al., 2018). Further research is needed to confirm whether these differences affect plant fitness. In this study, we discovered that the AHAS mutations detected (Pro197, Asp376, and Trp574 substitutions) are associated with reduced sensitivity and altered affinity to the tested herbicides, providing a structural basis for the differential resistance levels among mutation types via the ForteBio Octet system, and similar results were reported by *Chen* et al (Chen et al., 2023). However, the results of this study suggest that herbicide resistance in weeds may be related not only to the affinity between the target protein and herbicide but also to physiological processes, which need further research.

The conformation of sulfonylurea herbicides that bind to AHAS is affected by the aromatic nucleus and heterocycle of the herbicides (Garcia et al., 2017). Pyrazosulfuron-ethyl and bensulfuron-methyl are both sulfonylurea-type AHAS inhibitors. The former is composed of a pyrimidine ring, a urea bridge, and a five-membered heterocycle (pyrazole), whereas the latter consists of a pyrimidine ring, a urea bridge, and a benzene ring. The heterocycles are more easily hydrogenated than the benzene rings are. Therefore, pyrazosulfuron-ethyl may form hydrogen bonds more easily than bensulfuron-methyl. This may also have resulted from stronger hydrogen-bond interactions between pyrazosulfuron-ethyl and wild-type AHAS than between bensulfuron-methyl and wild-type AHAS in this study (Lonhienne et al., 2018). The Pro197Leu substitution may alter the local conformation of AHAS and weaken herbicide binding by reducing hydrogen bonds and changing hydrophobic interactions, thereby conferring resistance (Yang et al., 2018; Guo et al., 2022; Lonhienne et al., 2022; Madeira et al., 2023).

The Pro197Ser substitution alters the local conformation and physicochemical properties of the AHAS herbicide-binding pocket, reducing hydrogen bonding and hydrophobic interactions and thereby weakening herbicide binding. As a result, the herbicide is less able to stably inhibit AHAS, leading to resistance (Yang et al., 2018; Du et al., 2023). The Pro197Arg substitution may alter the local conformation of AHAS and weaken herbicide binding by changing the shape and physicochemical properties of the binding pocket, thereby reducing sensitivity to the herbicide (Xu et al., 2020; Guo et al., 2024). In addition, the Pro197Arg substitution may change hydrogen-bonding and hydrophobic interactions within the AHAS binding pocket, which can weaken inhibition by certain herbicides and lead to resistance (Jaña et al., 2014; Lonhienne et al., 2022).

The Pro197Ala and Pro197Ser substitutions confer resistance by altering the local conformation and physicochemical environment of the AHAS herbicide-binding channel, thereby reducing herbicide binding affinity. The Asp376 site is located within the channel cavity and is also part of the key resistance-related region of AHAS (Choe et al., 2015; Long et al., 2019; Porri et al., 2024). The Asp376Glu substitution may alter the side-chain geometry and local conformation of the AHAS binding channel, weakening herbicide binding and thereby conferring resistance (Garcia et al., 2017; Porri et al., 2024). The Trp574 substitution is located near the channel entrance and is important for shaping the AHAS substrate/herbicide channel. Because Trp has a bulky, hydrophobic indole side chain, it can influence herbicide binding by altering the local steric and interaction environment, rather than simply “forming strong π-π interactions” with the pyrimidine ring (McCourt et al., 2006). In this study, the Trp574Leu mutation replaces a bulky hydrophobic tryptophan with the smaller leucine, which can alter the geometry of the channel entrance and reduce herbicide binding affinity. As a result, the herbicide is less able to block substrate access to the catalytic center, contributing to resistance.

Pearson correlation analysis was performed to probe the quantitative relationships among binding affinity (pKD), enzymatic inhibition (pI_50_), whole-cell growth inhibition (pGR50), and docking scores. A statistically significant positive correlation was observed between p I_50_ and pGR_50_ (r = 0.992, p< 0.01), substantiating that the herbicidal activity is primarily mediated through AHAS target engagement, with minimal confounding from non-target-site resistance mechanisms in this panel. In contrast, the correlations between pKD and pIC50 (r = 0.424, p = 0.403), as well as between pKD and pGR_50_ (r = 0.414, p = 0.415), were positive but not statistically significant—a finding consistent with the non-competitive inhibition mode, wherein inhibitory potency is not strictly governed by ground-state binding affinity but rather by the efficiency of post-binding conformational arrest. Additionally, docking scores failed to correlate with any experimental parameters (|r| ≤ 0.355, all p ≥ 0.490), corroborating that static scoring functions are inadequate for quantitative potency prediction in this allosteric system and should be confined to qualitative binding mode analysis. Collectively, these analyses support the target-centric herbicidal action and provide correlative evidence for the non-competitive inhibition mechanism.

Recent structural work on AHAS has provided a useful framework for interpreting inhibitor binding and resistance. Crystal structures of plant AHAS showed that sulfonylureas and imidazolinones occupy overlapping but distinct regions of a channel leading to the active site, rather than mimicking substrates directly (Borota et al., 2026). Recent studies demonstrated that common resistance mutations, especially P197 and W574, reduce binding affinity and can disrupt time-dependent accumulative inhibition through mutation-driven conformational changes (Lonhienne et al., 2022). Additional crystal structures of AtAHAS with triasulfuron and related analogues confirmed that even closely related inhibitors can adopt subtly different binding modes, while still retaining strong accumulative inhibition (Cheng et al., 2024). Together with AHAS holoenzyme structures showing subunit communication in activation and feedback inhibition, these data support the view that inhibitor efficacy depends not only on local contacts but also on broader conformational effects (Lonhienne et al., 2020; Zhang et al., 2020).

## 5 Conclusion

In conclusion, different mutations in AHAS induce distinct structural alterations that reduce herbicide affinity and confer resistance. The differential resistance levels between the two herbicides reflect mutation-specific binding variations, underscoring the importance of conformational changes in determining target specificity and resistance intensity. Furthermore, the structural insights provided here lay a foundation for the rational design of next-generation herbicides capable of maintaining efficacy against mutated AHAS variants, potentially through tailored chemical modifications that accommodate mutation-induced structural shifts. These findings thus hold promise for both immediate field applications and long-term herbicide development.

## Data Availability

The original contributions presented in the study are included in the article/**Supplementary Material**. Further inquiries can be directed to the corresponding author.
