# Supplementary material for: AHAS structural changes caused by mutations result in differences in binding affinity and resistance to two herbicides in Cyperus difformis L
Source: Front Plant Sci. 2026 Sep 17;17:1932845. doi: 10.3389/fpls.2026.1932845 (PMC13631945; doi:10.3389/fpls.2026.1932845)
Supplement: Supplementary file 1 [file DataSheet1.pdf]

Appendix 1

Table S1. The various mutations that have occurred on the aforementioned reported types of weeds

Reference	Plant species	Mutation type
Heap 2025	*Conyza canadensis* L.	Pro197Ser/Ala and Asp376Glu
Larran et al. 2017	*Amaranthus palmeri* S. Watson	Trp574Leu
Liu et al. 2017	*Stellaria aquatica* L.	Pro197Ser
Yu et al. 2010	*Lolium perenne* L.	Pro197Ala/Gln/Arg/Tyr
Zhao et al. 2020	*Alopecurus aequalis* Sobol.	Trp574Leu
Fang et al. 2022	*Echinochloa crus-galli* (L.) P. Beauv	Phe206Leu
Li et al. 2013	*Raphanus raphanistrum* L.	Ala-122-Tyr, Pro-197-Ser, Asp-376-Glu and Trp-574-Leu
Han et al. 2012	*Raphanus raphanistrum* L.	Ala122Tyr
Ntoanidou et al. 2018	*Rapistrum rugosum* L.	Trp574 and Pro197
Palmieri et al. 2022	*Amaranthus palmeri* S. Watson	A122S, A205V, D376E, W574L and S653N
Zhang et al. 2017	*Descurainia sophia* L.	Pro197, Asp376 and Trp574
Yu et al. 2012	*Raphanus raphanistrum* L.	
Yang et al. 2018	*Descurainia sophia* L.	Pro197Leu/Ser, Asp376Glu, and Trp574Leu
Chen et al. 2023	*Eleusine indica* (L.) Gaertn.	Pro106Leu (P106L), Pro106Ser (P106S), and Thr102Ile + Pro106Ser

Table S2. The doses of bensulfuron methyl.

Populations	Bensulfuron methyl (g a.i. ha^{-1})
YX15-30	0, 0. 029, 0.058, 0. 234, 0. 468, 0. 938, 1.875
YX15-24-1, YX15-22-3, YX15-22-1	0, 30, 60, 120, 240, 480, 960, 1920
YX13-17-1	0, 120, 240, 480, 960, 1920, 3840, 7680
YX15-37-1	0, 480, 960, 1920, 3840, 7680

Table S3. The concentration of purified six AHAS.

Protein	260/280	Concentration (mg/ml)
Wild type	0.52	1.10
Asp376Glu	0.57	1.21
Pro197Leu	0.75	1.80
Pro197Ser	0.56	0.99
Pro197Arg	0.54	1.30
Trp574Leu	0.54	1.24

Purify the ALS analysis spectrum using AKTA advent

AHAS: YX15-30 (Wild type)
ALS1: YX 13-17-1(Asp376Glu)
ALS2: YX 15-22-1(Pro197Leu)
ALS3: YX 15-22-3 (Pro197Ser)
ALS4: YX 15-24-1(Pro197Arg)
ALS5: YX 15-37-1(Trp574Leu)

AHAS：

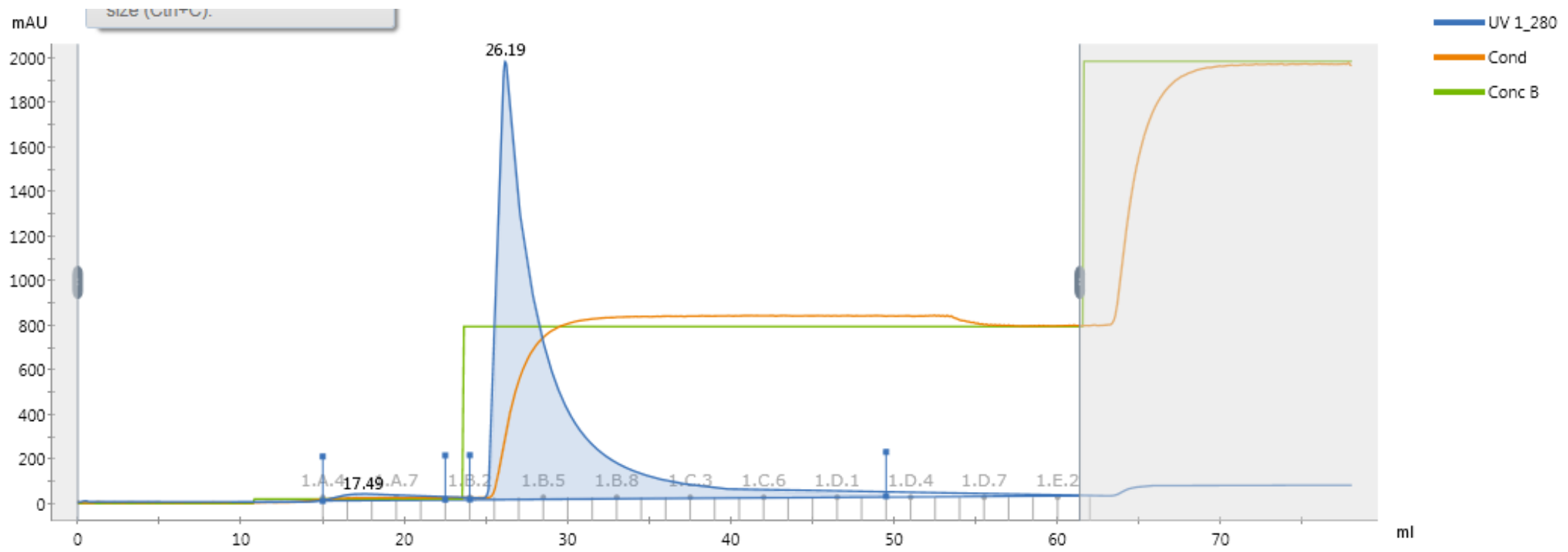

Peak Table - UV 1_280

Peak	Retention ml	Area ml*mAU	Area %	Ext coeff. mg ml⁻¹ cm⁻¹	Fraction(s)	Volume ml	Conductivity mS/cm
Peak A	17.488	150.3	2.41		1.A.4 - 1.A.8	7.500	28.77
Peak B	26.195	6085	97.59		1.B.2 - 1.D.2	25.503	40.26

ALS1：

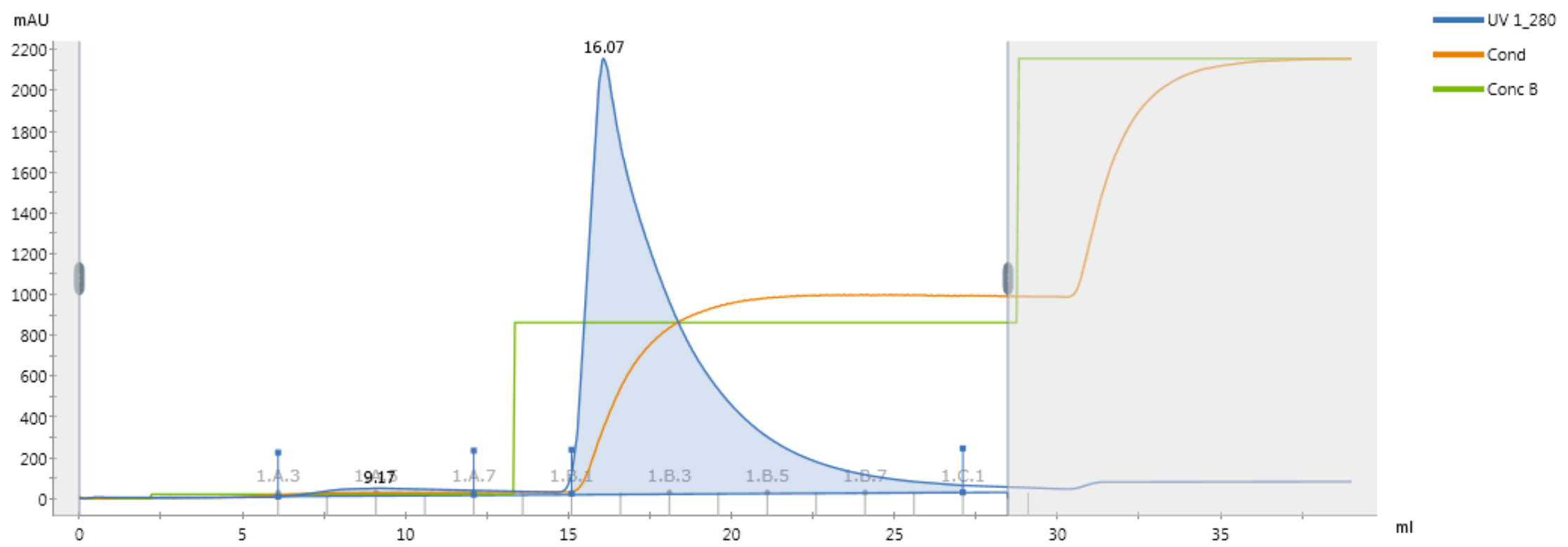

Peak Table - UV 1_280

Peak	Retention ml	Area ml*mAU	Area %	Ext coeff. mg ml⁻¹ cm⁻¹	Fraction(s)	Volume ml	Conductivity mS/cm
Peak A	9.165	145.0	2.26		1.A.3 - 1.A.6	6.002	28.87
Peak B	16.069	6279	97.74		1.B.1 - 1.B.8	12.001	40.82

ALS2：

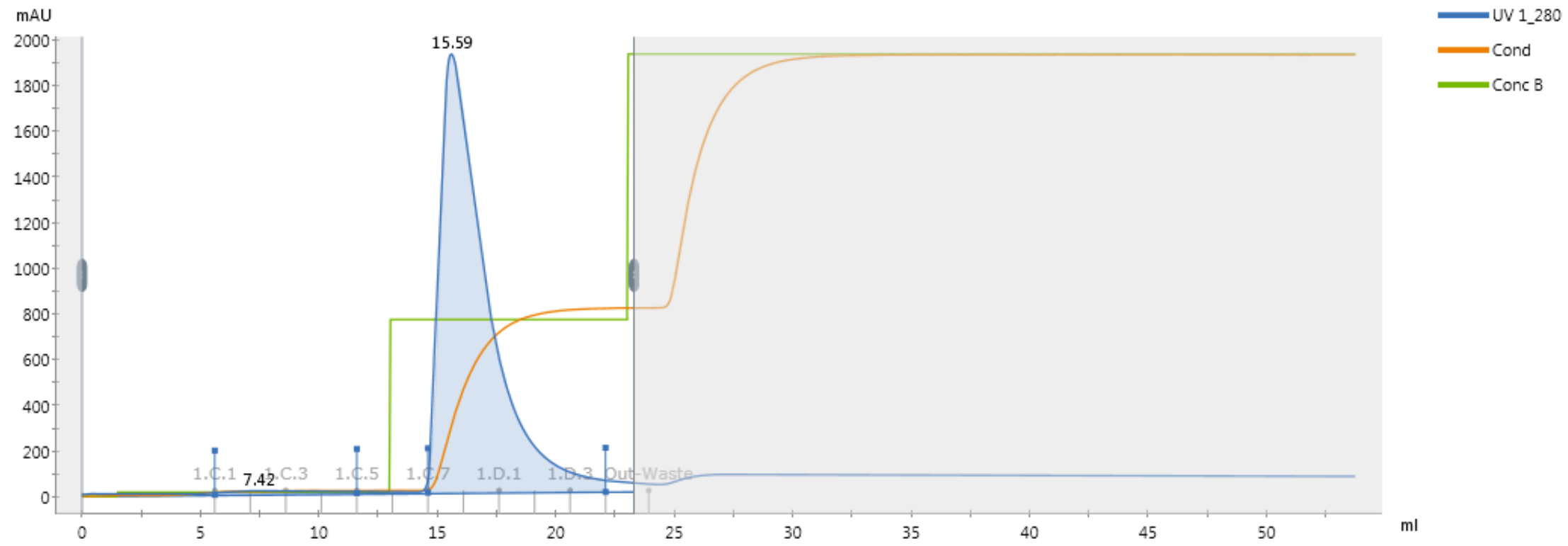

Peak Table - UV 1_280

Peak	Retention ml	Area ml*mAU	Area %	Ext coeff. mg ml⁻¹ cm⁻¹	Fraction(s)	Volume ml	Conductivity mS/cm
Peak A	7.421	77.97	1.75		1.C.1 - 1.C.4	5.999	28.91
Peak B	15.590	4382	98.25		1.C.7 - 1.D.3	7.498	39.01

ALS3：

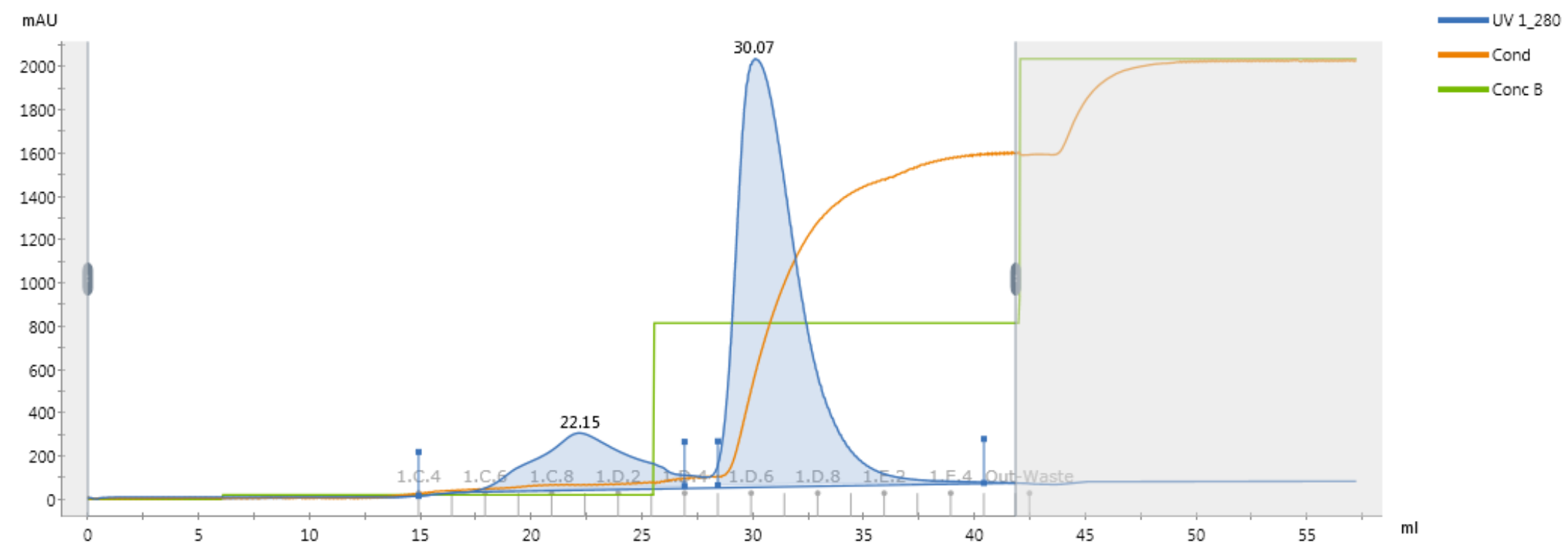

Peak Table - UV 1_280

Peak	Retention ml	Area ml*mAU	Area %	Ext coeff. mg ml⁻¹ cm⁻¹	Fraction(s)	Volume ml	Conductivity mS/cm
Peak A	22.155	1284	17.24		1.C.4 - 1.D.3	12.002	29.38
Peak B	30.070	6161	82.76		1.D.5 - 1.E.4	12.002	47.00

ALS4：

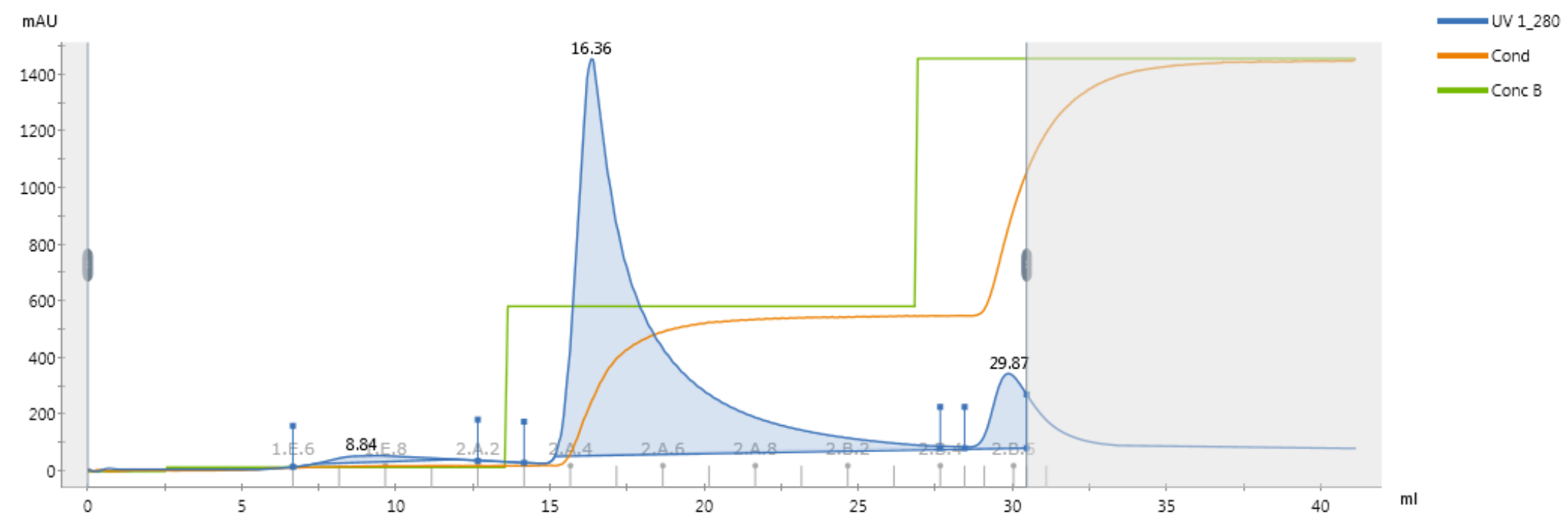

Peak Table - UV 1_280

Peak	Retention ml	Area ml*mAU	Area %	Ext coeff. mg ml⁻¹ cm⁻¹	Fraction(s)	Volume ml	Conductivity mS/cm
Peak A	8.838	66.35	1.77		1.E.6 - 2.A.1	5.998	28.93
Peak B	16.359	3408	90.68		2.A.3 - 2.B.3	13.500	38.04
Peak C	29.870	284.0	7.56		Waste(Frac) - 2.B.5	2.015	44.41

ALS5：

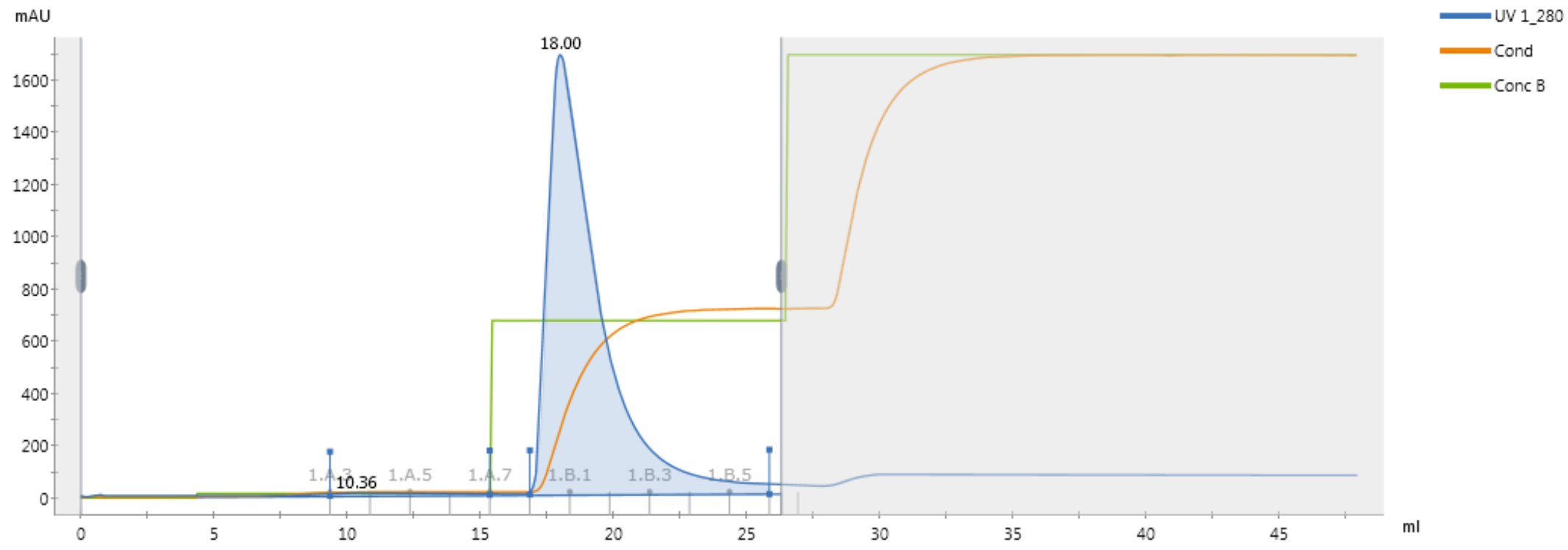

Peak Table - UV 1_280

Peak	Retention ml	Area ml*mAU	Area %	Ext coeff. mg ml⁻¹ cm⁻¹	Fraction(s)	Volume ml	Conductivity mS/cm
Peak A	10.362	48.40	1.29		1.A.3 - 1.A.6	6.002	29.04
Peak B	17.998	3711	98.71		1.A.8 - 1.B.5	8.999	39.42

SDS-PAGE gel result：

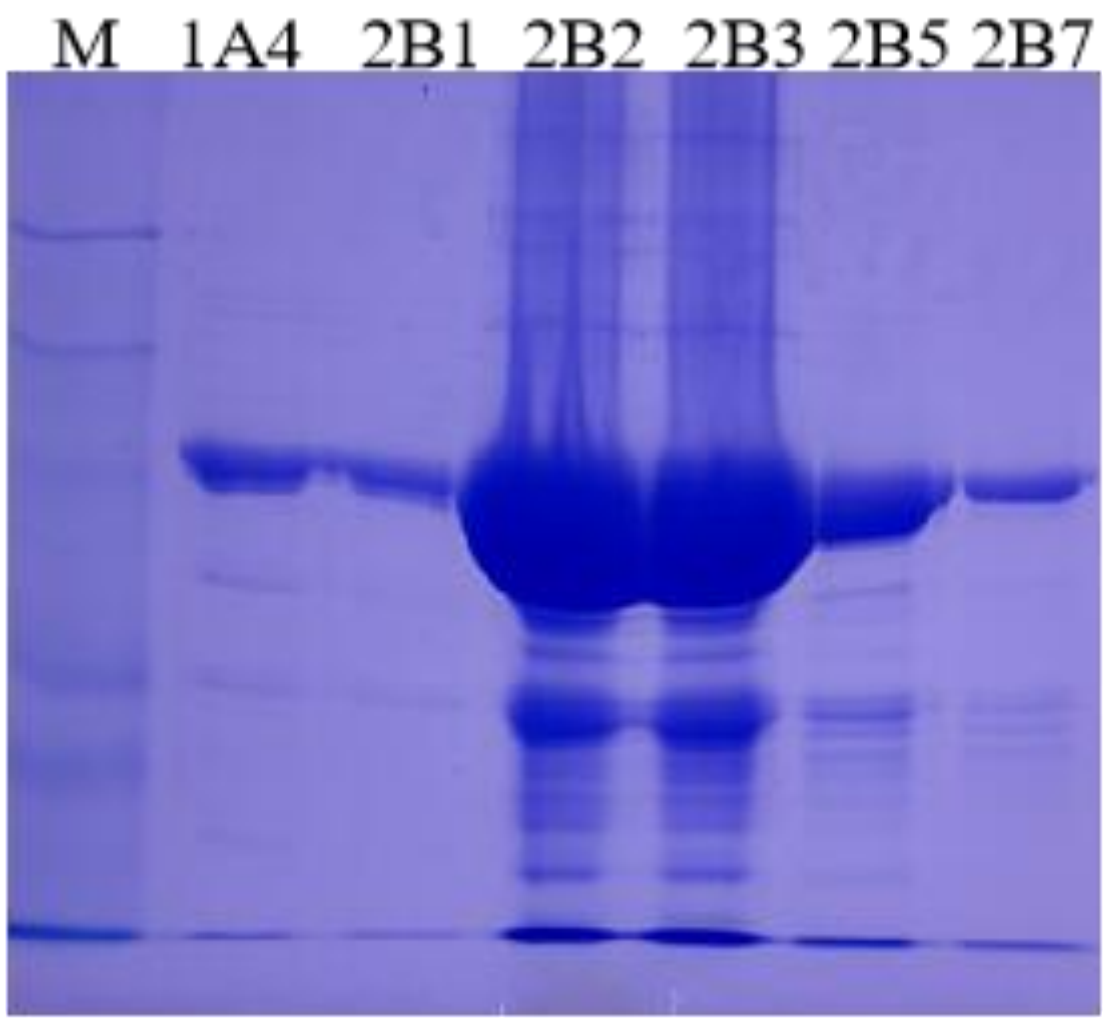

AHAS SDS-PAGE

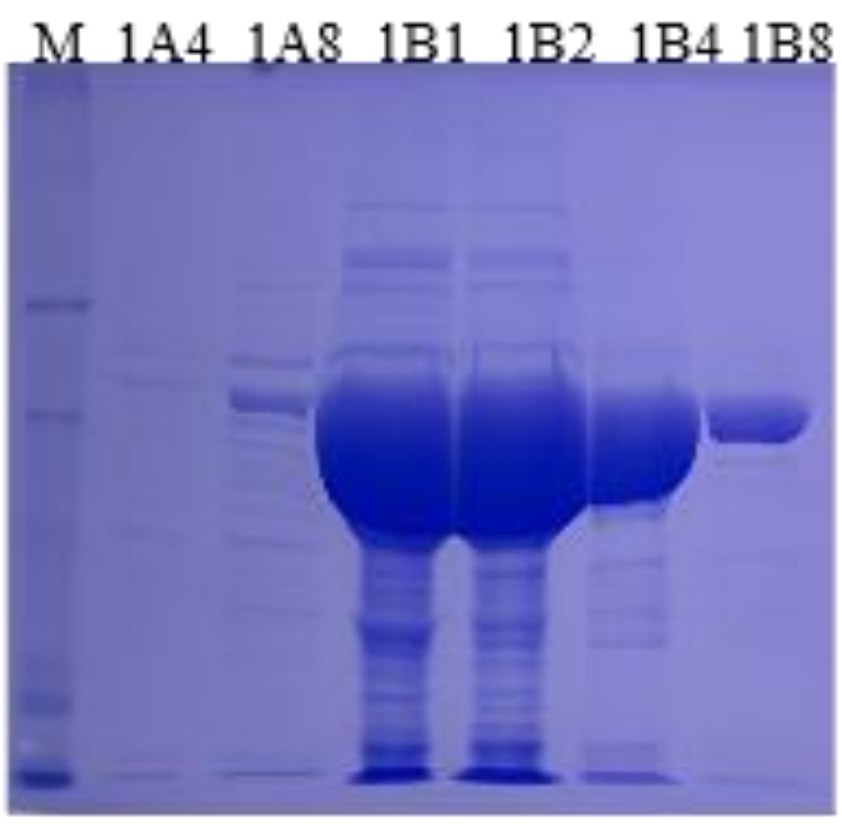

ALS1 SDS-PAGE

marker： 7-175kDa

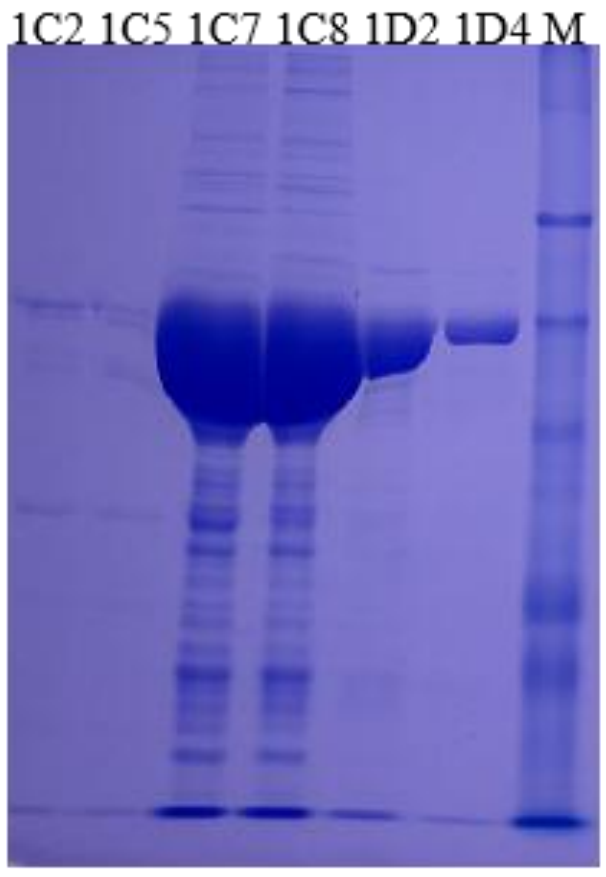

ALS2 SDS-PAGE

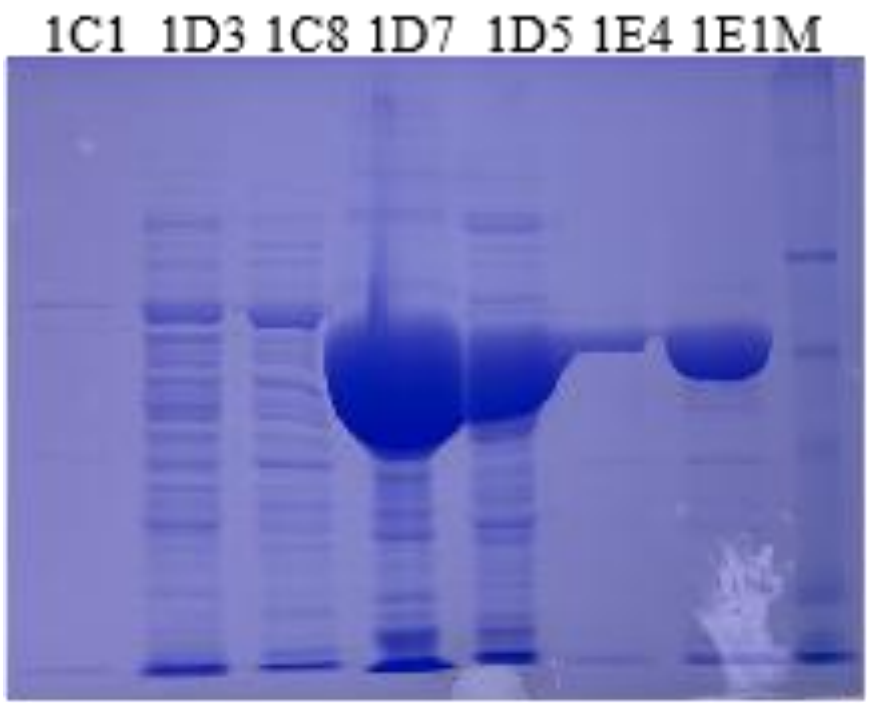

ALS3 SDS-PAGE

marker：7-175kDa

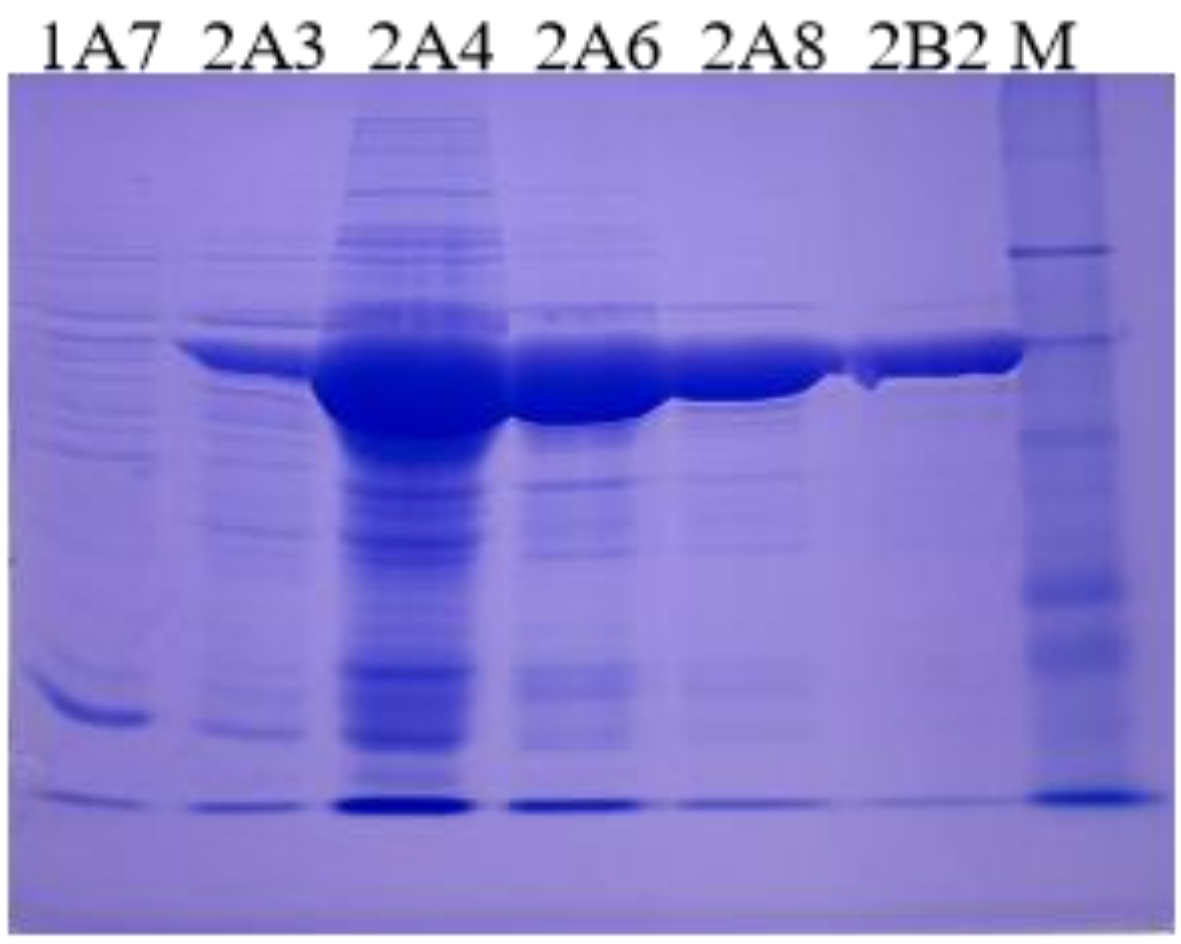

ALS4 SDS-PAGE

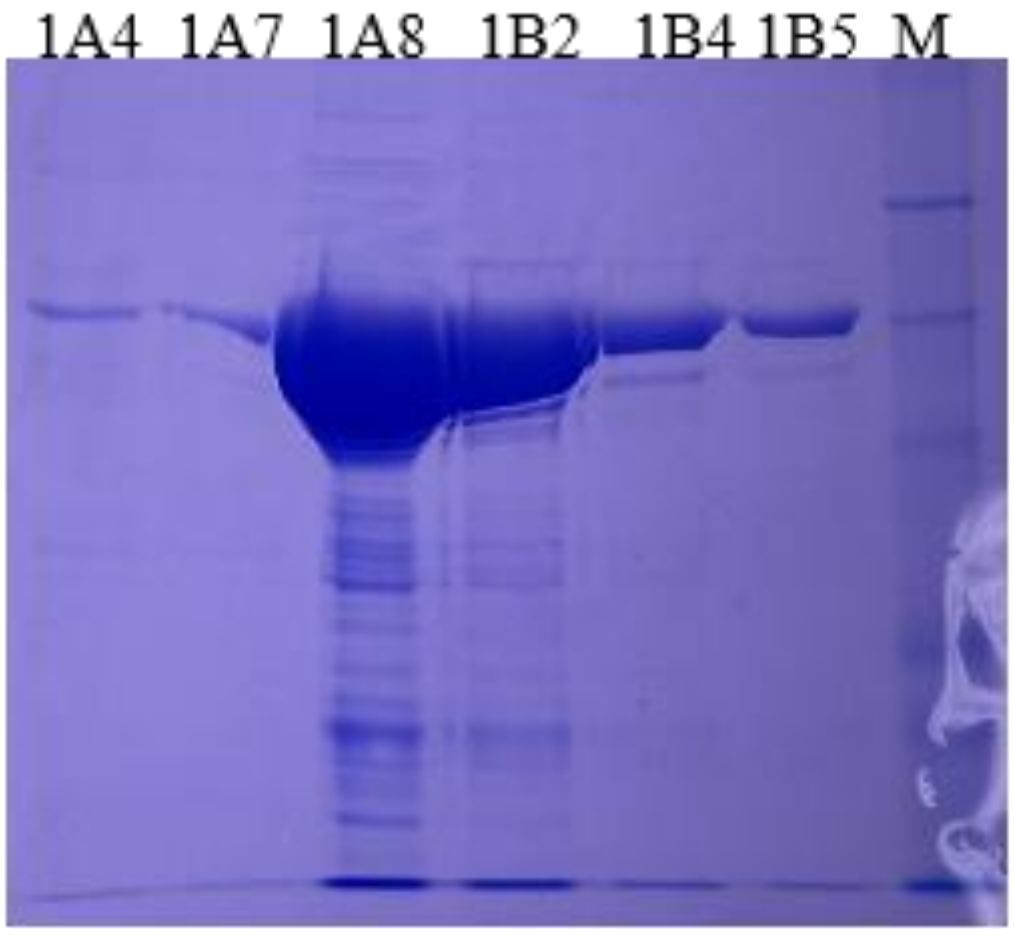

ALS5 SDS-PAGE

Analysis of ALS protein by SDS-PAGE after desalting

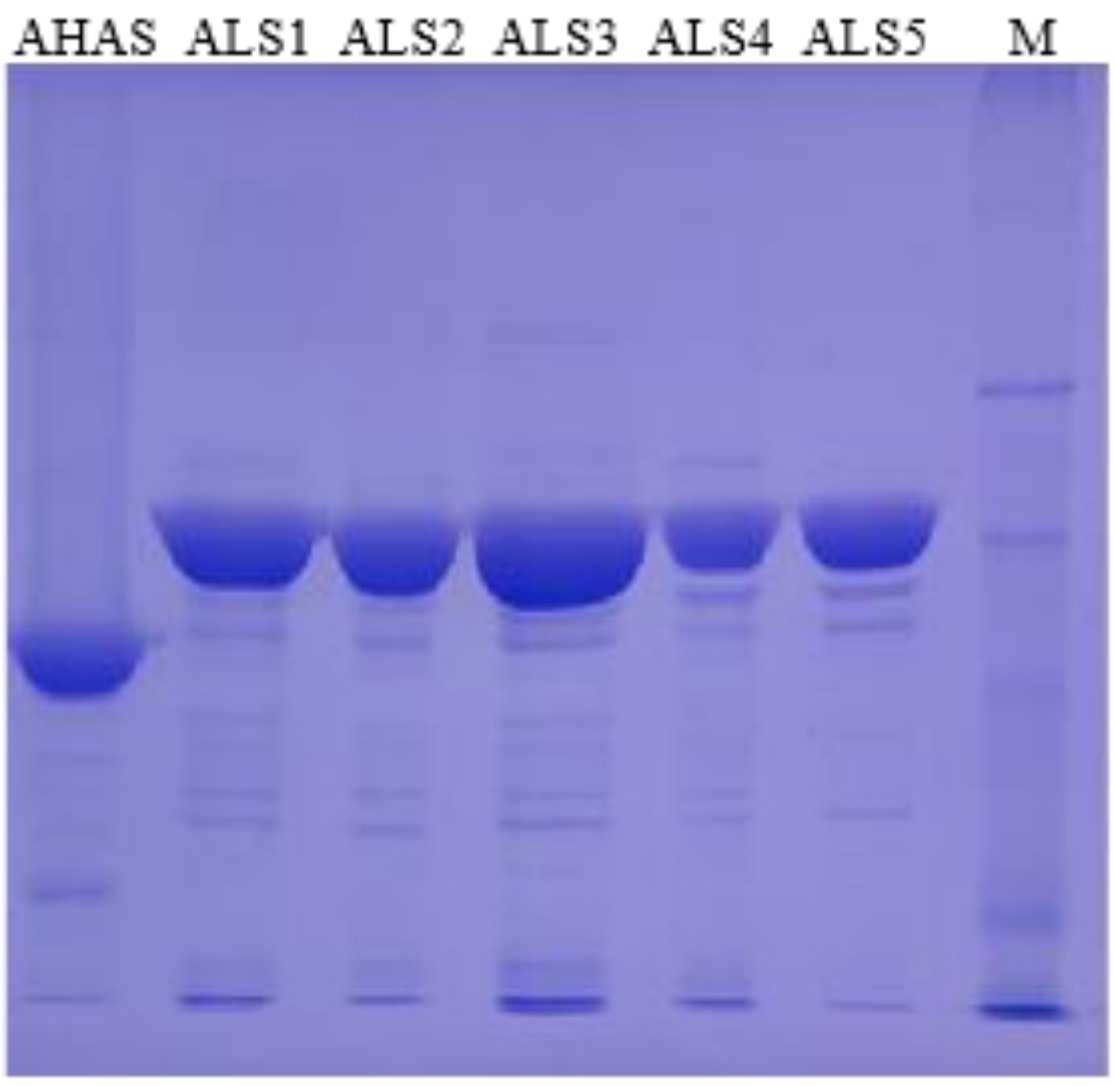

SDS-PAGE

Protein biotinylation coupling effect detection; Coupling effect detection

The machine detection parameters are as follows:

Assay Step Number	Step Data Name	Sensor Column	Sample Column	Step Type	Assay Time
1	Baseline2	1	1	Baseline	120
2	Baseline	1	1	Baseline	60
3	Association	1	6	Association	60

Detection of the affinity of the target protein to small molecules

The binding affinity between the biotinylated target protein and the small-molecule compounds was evaluated using the FortéBio Octet 96 system. Prior to the assay, the biosensors were pre-equilibrated by soaking in the appropriate buffer, and the diluted samples were subsequently loaded into the designated positions within the microplate. The measurement chamber was then sealed, and the instrument was programmed to execute the association and dissociation phases sequentially. The detection parameters employed in the assay are outlined as follows:

N	Data Name	Assay Time	Flow Rate	Step Type Name	Step Type
1	Baseline	60	1000	Baseline	2
2	Association	60	1000	Association	0
3	Quench	60	1000	Quench	1
4	Baseline2	120	1000	Baseline	2

Assay Step Number	Step Data Name	Sensor Column	Sample Column	Step Type	Assay Time
1	Baseline2	1	1	Baseline	120
2	Baseline	1	1	Baseline	60
3	Association	1	6	Association	60
4	Quench	1	6	Quench	60

Biosensors and diluted samples were loaded into the corresponding wells of the FortéBio Octet 96 instrument. Following closure of the lid, the assay protocol was initiated to carry out the affinity detection.

N	Data Name	Assay Time	Flow Rate	Step Type Name	Step Type
1	Baseline	60	1000	Baseline	2
2	Association	60	1000	Association	0
3	Dissociation	180	1000	Dissociation	1
4	Baseline2	120	1000	Baseline	2

Assay Step Number	Step Data Name	Sensor Column	Sample Column	Step Type	Assay Time
1	Baseline2	1	1	Baseline	120
2	Baseline	1	1	Baseline	60
3	Association	1	6	Association	60
4	Dissociation	1	1	Dissociation	180
5	Baseline	1	2	Baseline	60
6	Association	1	7	Association	60
7	Dissociation	1	2	Dissociation	180
8	Baseline	1	3	Baseline	60
9	Association	1	8	Association	60
10	Dissociation	1	3	Dissociation	180
11	Baseline	1	4	Baseline	60
12	Association	1	9	Association	60
13	Dissociation	1	4	Dissociation	180
14	Baseline	1	5	Baseline	60
15	Association	1	10	Association	60

16	Dissociation	1	5	Dissociation	180
17	Baseline2	2	1	Baseline	120
18	Baseline	2	1	Baseline	60
19	Association	2	6	Association	60
20	Dissociation	2	1	Dissociation	180
21	Baseline	2	2	Baseline	60
22	Association	2	7	Association	60
23	Dissociation	2	2	Dissociation	180
24	Baseline	2	3	Baseline	60
25	Association	2	8	Association	60
26	Dissociation	2	3	Dissociation	180
27	Baseline	2	4	Baseline	60
28	Association	2	9	Association	60
29	Dissociation	2	4	Dissociation	180
30	Baseline	2	5	Baseline	60
31	Association	2	10	Association	60
32	Dissociation	2	5	Dissociation	180

The details related to modeling are as follows:

Table S4. Information of modeling

Template	Mutations	Sequence Identity	Cover/%	Resolution/Å
3e9y.1	YX15-30 (wild type)	78.45%	62-639 (90%)	3.00 Å
	YX15-22-1 (Pro197Leu)	78.41%	63-639 (90%)	3.00 Å
	YX15-22-3 (Pro197Ser)	78.28%	62-639 (90%)	3.00 Å
	YX 15-24-1 (Pro197Arg)	78.28%	62-639 (90%)	3.00 Å
	YX 13-17-1 (Asp376Glu)	78.28%	62-639 (90%)	3.00 Å
	YX 15-37-1 (Trp574Leu)	78.28%	62-639 (90%)	3.00 Å

Table S5 Evaluation results by Verify, Procheck and Errat.

**Mutations**	**Verify3D (3D-1D≥0.2)**	**Procheck**	**Errat**
Wild type-YX15-30	88.58%	90.1%, 9.4%, 0.4%	96.8393
Asp376Glu-YX13-17-1	89.88%	90.8%, 8.8%, 0.4%	98.7858
Pro197Leu-YX15-22-1	87.09%	89.5%, 10.1%, 0.4%	96.3940
Pro197Ser-YX15-22-3	88.93.%	90.0%, 9.6%, 0.4%	96.0492
Pro197Arg-YX15-24-1	88.32%	90.0%, 9.6%, 0.4%	96.1370
Trp574Leu- YX15-37-1	87.89%	89.9%, 9.7%, 0.4%	97.3661

YX13-17-1

Template	Seq Identity	Oligo-state	QSQE	Found by	Method	Resolution	Seq Similarity	Range	Coverage	Description
3e9y.1.A	78.28	homo-dimer	0.89	HHblits	X-ray	3.00Å	0.54	62 - 639	0.90	Acetolactate synthase, chloroplastic

YX 15-22-1

Template	Seq Identity	Oligo-state	QSQE	Found by	Method	Resolution	Seq Similarity	Range	Coverage	Description
3e9y.1.A	78.41	homo-dimer	0.88	HHblits	X-ray	3.00Å	0.54	63 - 639	0.90	Acetolactate synthase, chloroplastic

YX 15-22-3

Template	Seq Identity	Oligo-state	QSQE	Found by	Method	Resolution	Seq Similarity	Range	Coverage	Description
3e9y.1.A	78.28	homo-dimer	0.89	HHblits	X-ray	3.00Å	0.54	62 - 639	0.90	Acetolactate synthase, chloroplastic

YX 15-24-1

Template	Seq Identity	Oligo-state	QSQE	Found by	Method	Resolution	Seq Similarity	Range	Coverage	Description
3e9y.1.A	78.28	homo-dimer	0.89	HHblits	X-ray	3.00Å	0.54	62 - 639	0.90	Acetolactate synthase, chloroplastic

YX 15-37-1

Template	Seq Identity	Oligo-state	QSQE	Found by	Method	Resolution	Seq Similarity	Range	Coverage	Description
3e9y.1.A	78.28	homo-dimer	0.89	HHblits	X-ray	3.00Å	0.54	62 - 639	0.90	Acetolactate synthase, chloroplastic

YX 15-30

Template	Seq Identity	Oligo-state	QSQE	Found by	Method	Resolution	Seq Similarity	Range	Coverage	Description
3e9y.1.A	78.45	homo-dimer	0.89	HHblits	X-ray	3.00Å	0.54	62 - 639	0.90	Acetolactate synthase, chloroplastic
