# Supplementary material for: AHAS structural changes caused by mutations result in differences in binding affinity and resistance to two herbicides in Cyperus difformis L
Source: Front Plant Sci. 2026 Sep 17;17:1932845. doi: 10.3389/fpls.2026.1932845 (PMC13631945; doi:10.3389/fpls.2026.1932845)
Supplement: Supplementary file 2 [file DataSheet10.pdf]

PROCHECK

Ramachandran Plot

saves

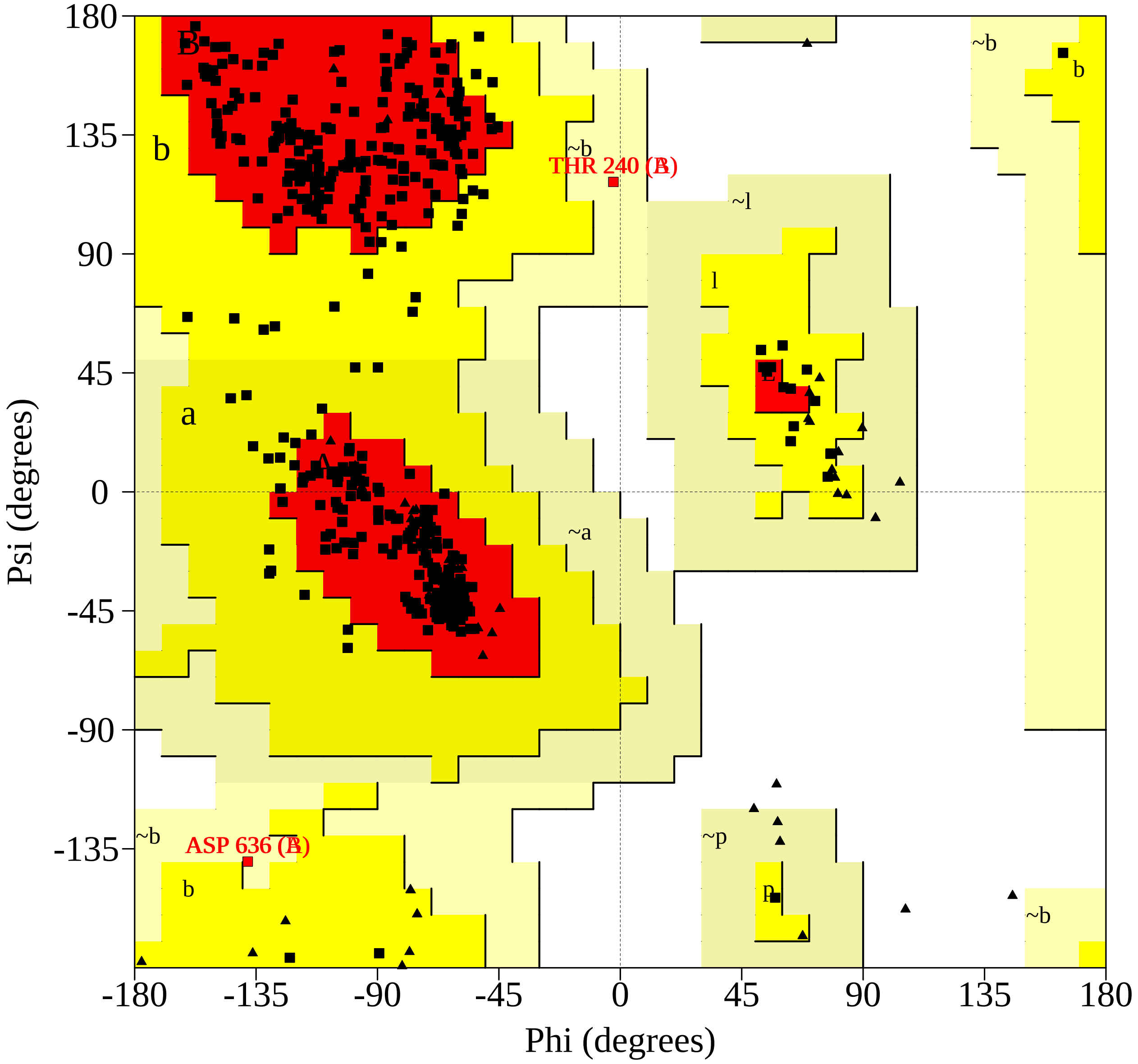

Plot statistics

Residues in most favoured regions [A,B,L]	878	90.0%
Residues in additional allowed regions [a,b,l,p]	94	9.6%
Residues in generously allowed regions [~a,~b,~l,~p]	4	0.4%
Residues in disallowed regions	0	0.0%
	----	------
Number of non-glycine and non-proline residues	976	100.0%
Number of end-residues (excl. Gly and Pro)	10	
Number of glycine residues (shown as triangles)	102	
Number of proline residues	74	

Total number of residues	1162	

Based on an analysis of 118 structures of resolution of at least 2.0 Angstroms
and R-factor no greater than 20%, a good quality model would be expected
to have over 90% in the most favoured regions.
