# Supplementary material for: AHAS structural changes caused by mutations result in differences in binding affinity and resistance to two herbicides in Cyperus difformis L
Source: Front Plant Sci. 2026 Sep 17;17:1932845. doi: 10.3389/fpls.2026.1932845 (PMC13631945; doi:10.3389/fpls.2026.1932845)
Supplement: Supplementary file 6 [file DataSheet2.pdf]

Editing Certificate

This document certifies that the manuscript

AHAS Structural Changes Caused by Mutations Result in Differences in Binding Affinity and Resistance to Two Herbicides in Cyperus difformis L.

prepared by the authors

Xiaotong Guo, Xiangju Li , Jingchao Chen , Linzhi Bai , Haiyan Yu, and Hailan Cui

was edited for proper English language, grammar, punctuation, spelling, and overall style by one or more of the highly qualified English speaking editors at AJE.

This certificate was issued on **July 28, 2025** and may be verified on the AJE website using the verification code **0408-7924-08F7-C977-AD05**.

Neither the research content nor the authors' intentions were altered in any way during the editing process. Documents receiving this certification should be English-ready for publication; however, the author has the ability to accept or reject our suggestions and changes. To verify the final AJE edited version, please visit our verification page at aje.com/certificate. If you have any questions or concerns about this edited document, please contact AJE at support@aje.com.

AJE provides a range of editing, translation, and manuscript services for researchers and publishers around the world. For more information about our company, services, and partner discounts, please visit aje.com.
