# Supplementary material for: AHAS structural changes caused by mutations result in differences in binding affinity and resistance to two herbicides in Cyperus difformis L
Source: Front Plant Sci. 2026 Sep 17;17:1932845. doi: 10.3389/fpls.2026.1932845 (PMC13631945; doi:10.3389/fpls.2026.1932845)
Supplement: Supplementary file 8 [file DataSheet4.pdf]

PROCHECK

# Ramachandran Plot

## saves

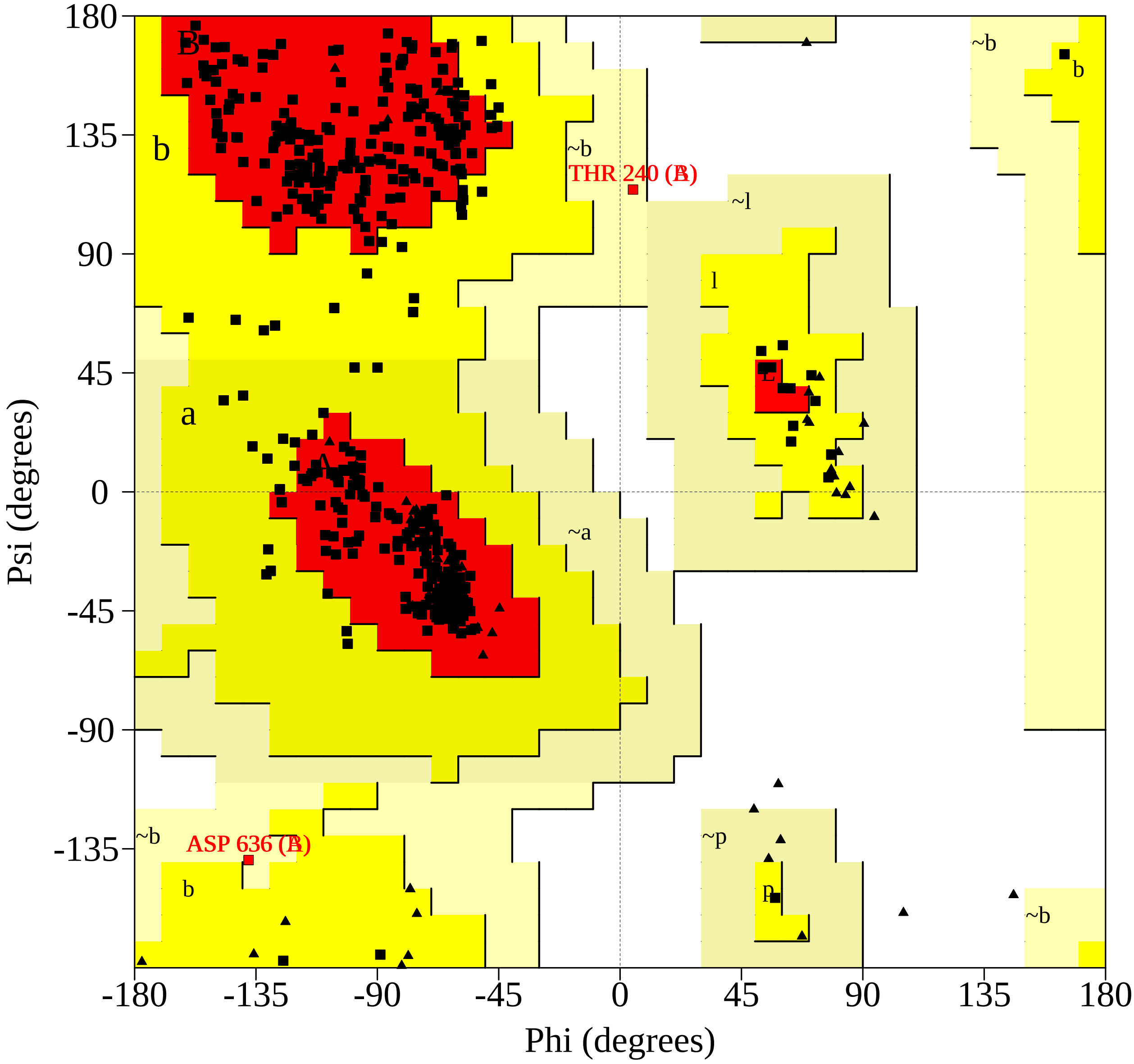


Plot statistics

| | | |
|---|---|---|
| Residues in most favoured regions [A,B,L] | 884 | 90.8% |
| Residues in additional allowed regions [a,b,l,p] | 86 | 8.8% |
| Residues in generously allowed regions [~a,~b,~l,~p] | 4 | 0.4% |
| Residues in disallowed regions | 0 | 0.0% |
| | ---- | ------ |
| Number of non-glycine and non-proline residues | 974 | 100.0% |
| Number of end-residues (excl. Gly and Pro) | 10 | |
| Number of glycine residues (shown as triangles) | 102 | |
| Number of proline residues | 76 | |
| | ---- | |
| Total number of residues | 1162 | |

Based on an analysis of 118 structures of resolution of at least 2.0 Angstroms and R-factor no greater than 20%, a good quality model would be expected to have over 90% in the most favoured regions.
