# Supplementary material for: AHAS structural changes caused by mutations result in differences in binding affinity and resistance to two herbicides in Cyperus difformis L
Source: Front Plant Sci. 2026 Sep 17;17:1932845. doi: 10.3389/fpls.2026.1932845 (PMC13631945; doi:10.3389/fpls.2026.1932845)
Supplement: Supplementary file 11 [file DataSheet7.pdf]

Program: ERRAT2
File: 15-22-3.pdb
Chain#:A
Overall quality factor**: 96.049

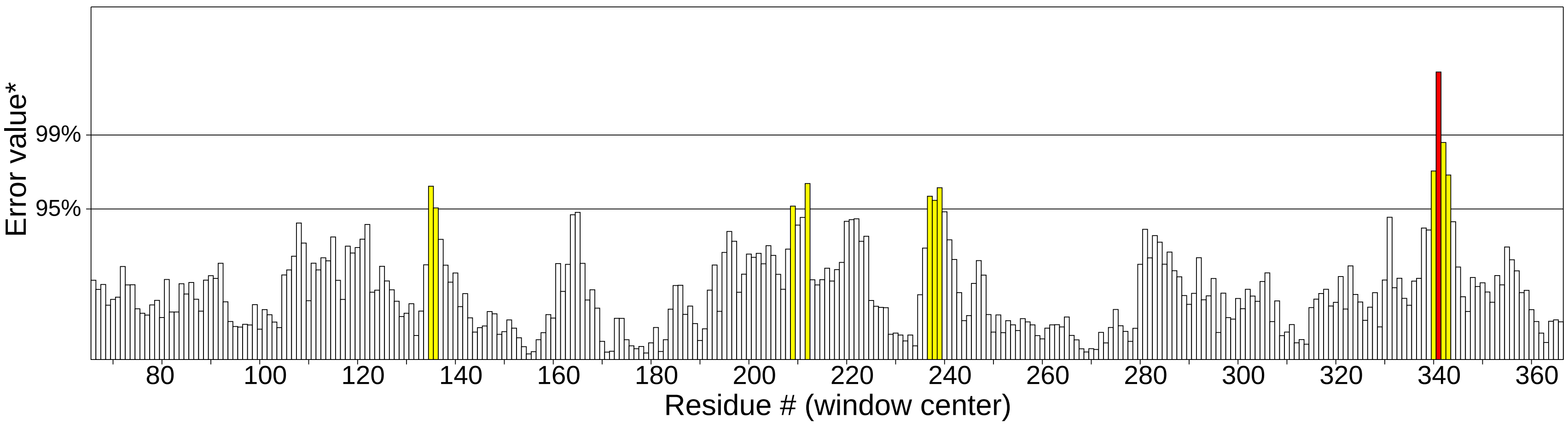

*On the error axis, two lines are drawn to indicate the confidence with which it is possible to reject regions that exceed that error value.

**Expressed as the percentage of the protein for which the calculated error value falls below the 95% rejection limit. Good high resolution structures generally produce values around 95% or higher. For lower resolutions (2.5 to 3A) the average overall quality factor is around 91%.

Program: ERRAT2
File: 15-22-3.pdb
Chain#:A
Overall quality factor**: 96.049

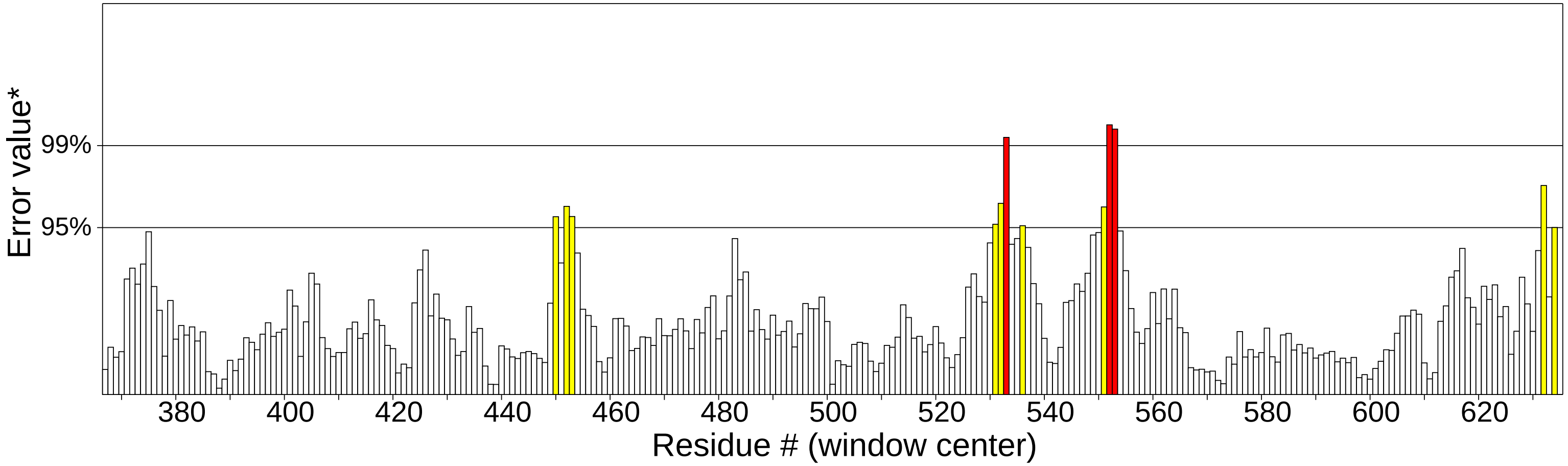

*On the error axis, two lines are drawn to indicate the confidence with which it is possible to reject regions that exceed that error value.

**Expressed as the percentage of the protein for which the calculated error value falls below the 95% rejection limit. Good high resolution structures generally produce values around 95% or higher. For lower resolutions (2.5 to 3A) the average overall quality factor is around 91%.

Program: ERRAT2
File: 15-22-3.pdb
Chain#:B
Overall quality factor**: 96.049

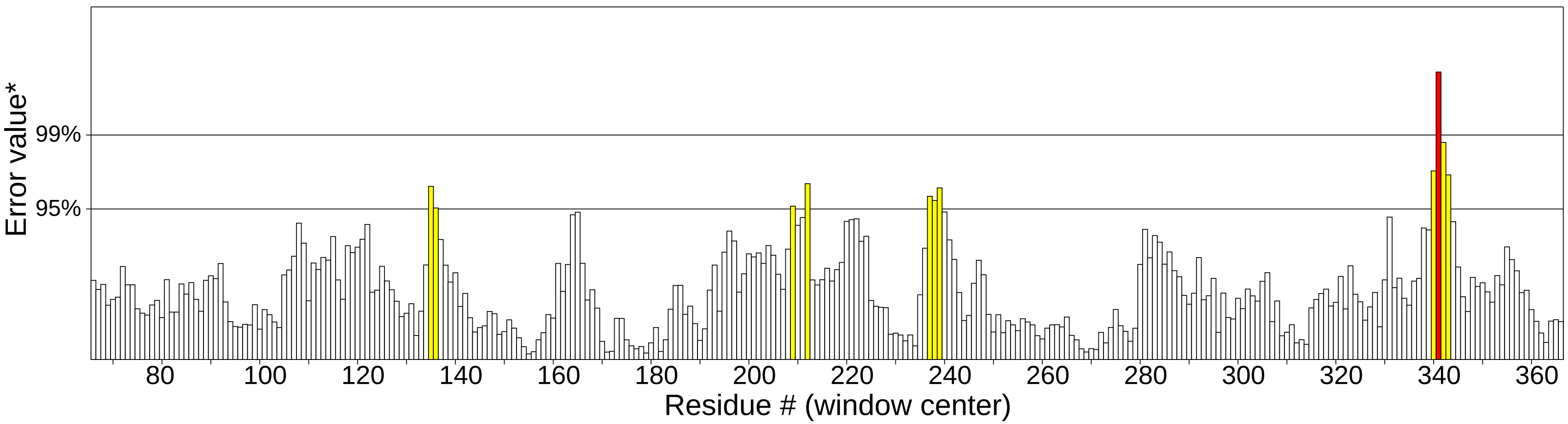

*On the error axis, two lines are drawn to indicate the confidence with which it is possible to reject regions that exceed that error value.

**Expressed as the percentage of the protein for which the calculated error value falls below the 95% rejection limit. Good high resolution structures generally produce values around 95% or higher. For lower resolutions (2.5 to 3A) the average overall quality factor is around 91%.

Program: ERRAT2
File: 15-22-3.pdb
Chain#:B
Overall quality factor**: 96.049

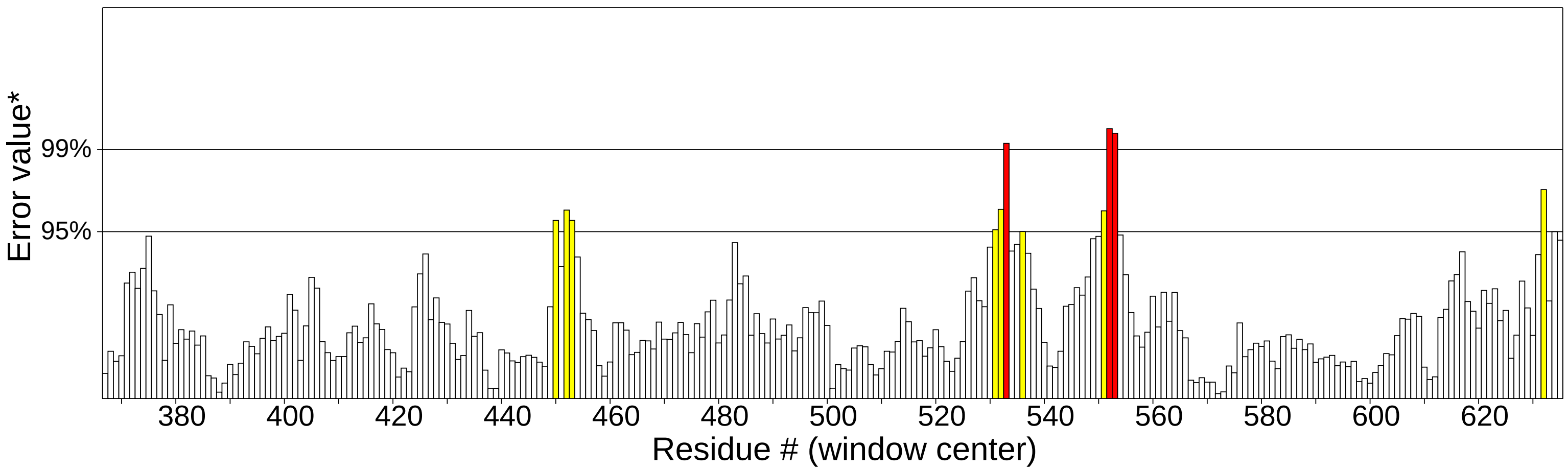

*On the error axis, two lines are drawn to indicate the confidence with which it is possible to reject regions that exceed that error value.

**Expressed as the percentage of the protein for which the calculated error value falls below the 95% rejection limit. Good high resolution structures generally produce values around 95% or higher. For lower resolutions (2.5 to 3A) the average overall quality factor is around 91%.
