# Supplementary material for: AHAS structural changes caused by mutations result in differences in binding affinity and resistance to two herbicides in Cyperus difformis L
Source: Front Plant Sci. 2026 Sep 17;17:1932845. doi: 10.3389/fpls.2026.1932845 (PMC13631945; doi:10.3389/fpls.2026.1932845)
Supplement: Supplementary file 17 [file Table4.doc]

>24

1	ATGGCTACCC CTCTCCACAC CAGCGCCGCC ACCCCCACTC CCTCCGTCCG CCGCTTCACA

61	CCTCCCCTCC TCACCCCCAT TTTCCTATCC AAGCACTCCA AACCCTCCTC TTTCTCCCTC

121	CACGCCCTCA GTCATACCAC CGCCCCCCCT CTCACTGCGC CCCCTCAGCG CCCCTTTCGC

181	AATCTCTCCC CCACCGACCC CCGCAAGGGT GCCGATATAC TCGTCGAGGC GCTCGAGAGA

241	CAAGGTGTCA CCGATGTCTT CGCCTATCCA GGCGGAGCCT CCATGGAAAT TCACCAAGCC

301	CTTACGAGGT CTCCTGTCAT AGACAATCAC CTCTTGAGGC ATGAACAGGG AGAGTCCTTC

361	GCGGCCTCCG GCTATGCGCG CTCTACCGGC AAGGCTGGGG TTTGTGTGGC CACGTCCGGG

421	CCCGGAGCAA CGAATCTCGT CTCTGCTCTT GCTGATGCAT TGCTTGATTC AGTTCCTATG

481	GTCGCAATCA CTGGACAGGT CCGTCGTCGC ATGATTGGTA CCGATGCCTT TCAAGAGACC

541	CCAATTGTTG AAGTGACTCG TTCCATAACA AAGCACAACT ATCTTGTACT CGACGTAGAT

601	GACATACCTC GCATCATCAA AGAGGCATTT TTCTTGGCCA CTTCAGGCCG TCCGGGACCC

661	GTTTTGGTCG ACATTCCAAA GGACATTCAA CAACAATTGG CTGTACCAGT GTGGGACACA

721	CCAATGCGCC TTCCAGGATA CACCTCTCGC CTGCCAAAGC AACCTGAAGA CAACCAGCTT

781	GATCAGATAA TCCGTCTTGT TTCTGAATCA AAGCGGCCAG TGTTGTATGT AGGAGGCGGA

841	TGTGCCAACT CGGGTGCAGA GTTGAAACGA TTTGTGGAGC TAACGGGTAT ACCTGTTACT

901	ACTACCTTGA TGGGTCTTGG TAACTACCCT AGCAATGATG AGCTGTCTCT ACGCATGTTG

961	GGTATGCATG GCACTGTATA TGCAAATTAT GCAGTTGACA AAGCAGATCT GTTGTTAGCC

1021	TTTGGGGTGA GATTTGATGA TCGTGTTACT GGAAAGCTTG AGGCATTTGC TAGTCGCTCT

1081	AAAATTGTGC ACATAGATAT TGACCCAGCT GAAATTGGCA AGAATAAACA ACCACACGTG

1141	TCGATCTGTG CAGACGTCAA ACCTGCTTTG CAAGGCATGA ACCAAATACT GGAGTCTAGT

1201	GGGGTGCACA AAAAATTGGA TTTTTCTAGT TGGAGGGCTG AACTGGATGA GCAAAAGAAA

1261	ACATACCCAT TAAGCTACAA AACTTTTGGA GAGGAAATTC CCCCACAGTA CGCCATCCAG

1321	GTGCTTGATG AATTGACCAA CGGAGAAGCA ATTATAAGCA CAGGTGTCGG TCAGCACCAA

1381	ATGTGGGCTG CACAGTATTA CAACTATAAG AGACCTCGTC AGTGGCTTTC CAGTTCAGGT

1441	TTGGGTGCCA TGGGTTTCGG GTTACCTGCA GCTGCTGGGG CTGCAGTGGG AAACCCGGGT

1501	GTTACTGTGG TTGACATAGA CGGTGATGGT TCCTTCCTGA TGAATATCCA AGAGCTTGCC

1561	ATGATAAAGG TGGAGAACCT ACCTGTCAAG ACCATGGTGT TGAACAACCA ACACTTGGGA

1621	ATGGTGGTAC AATGGGAGGA CCGGTTTTAC AAGGCCAACC GGGCACACAC CTACTTAGGT

1681	AACCCGGCTA ATGAGGAGCA GATATATCCT GATTTTGTCA AGATAGCTGA AGGTTTCGGT

1741	GTACCTGCAG CACGTGTTAC AAGGAGGAGT GAGGTCCGAG AGGCAGTGAG GAAAATGTTG

1801	GATACACCAG GCCCATACCT GCTGGATGTG ATCGTACCGC ATCAGGAGCA TGTCTTGCCA

1861	ATGATTCCAA GTGGAGGGGC ATTCAAGGAT ATGATCCTCG ATGGAGATGG CCGGACTGTG

1921	TACTAG
